# A multi-scale observation and crack statistics based method for analyzing failure mechanism of pre-flawed rock under true triaxial stress

**DOI:** 10.1371/journal.pone.0323809

**Published:** 2025-05-23

**Authors:** Bangxiang Li, Xiaoqun Wang, Hongbo Zhao, Dongyang Xu, Guanhua Wang, Tian Su, Xuelei An

**Affiliations:** 1 School of Civil Engineering and Geomatics, Shandong University of Technology, Zibo, China; 2 Shandong Energy Group, Jinan, China; 3 Institute of Rock and Soil Mechanics, Chinese Academy of Sciences, Wuhan, China; 4 Shandong Survey and Design Institute of Water Conservancy Co., Ltd, Jinan, China; University of Sharjah, UNITED ARAB EMIRATES

## Abstract

Understanding the failure mechanism of the rock mass under the general stress state is of great importance for the safe constructions of the underground engineering. Here, a series of true triaxial fracture tests on the intact and pre-flawed sandstones are conducted. The failure modes of the sandstones are analyzed, and the multi-scale fracture characteristics of the basic types of cracks are identified. Moreover, a multi-scale observation and crack statistics based method for analyzing the failure mechanism of the rock is proposed, and the influences of the stress state and the pre-existing flaw on the rock failure mechanism are investigated. The results indicate that the rock failure mode is controlled by the true triaxial stress state and the pre-existing flaw. The crack quantities in the pre-flawed rocks are nearly always more than those in the intact rock. which indicates that pre-existing flaw has a significant promoting effect on the crack initiation and development. 7 types of basic crack in the rock failure modes are summarized. Based on the multi-scale fracture characteristics, the fracture mechanisms of the basic types of cracks are identified, and the fracture mechanisms of the seven basic type cracks are identified and divided into four categories. The quantity statistics of the cracks corresponding to different fracture mechanisms show that the rise of *σ*_3_ can significantly reduce the percentage of the shear cracks, while the rise of *σ*_2_ conduces to the increase of the percentage of the tensile crack. The pre-existing flaw has a promoting effect on the initiation of the tensile crack, however, the true triaxial stress is the decisive factor controlling the rock failure mechanism. In the discussion, the size effect of rock fracture and the correlation between true triaxial test and engineering application are analyzed. This work contributes to an improved understanding of the failure mechanism of rock and a potential means by which to guide the design and construction of underground engineering.

## 1. Introduction

Recently, the deep underground space has been the important land resource which various countries are racing to explore and develop [1]. The deep underground rocks are generally hosted in the true triaxial high stress (*σ*_1_ > *σ*_2_ > *σ*_3_) [2], and extensively developed joints and flaws [3–6]. The engineering excavation will initiate the redistribution of the in-situ stress. The loading and unloading stress can easily induce the propagations of the joints and the flaws, leading to engineering disaster such as rock burst, collapse, et al., and causing severe casualties and property loss. Hence, investigating the failure mechanism of the rock mass under the true triaxial stress is of great significance for guaranteeing the safety construction of the deep underground engineering.

To simulate the stress environment in deep underground, many scholars developed true triaxial apparatus, and carried out a mass of true triaxial fracture tests on rocks. In the aspect of the macro fracture characteristic [2,7–12], Feng et al. investigated the influences of the intermedium and minimum principal stresses (*σ*_2_, *σ*_3_) on the failure mode of the rock, and found *σ*_3_ had a greater influence than *σ*_2_ [13]. He et al. [14–17], Li et al. [18–20], Su et al. [21–24] conducted a series of true triaxial rockburst experiments on hard rocks, and studied the factors affecting the ejection features of the rockburst. Lu et al. researched the failure modes of coal and sandstone under different true triaxial loading paths [25]. Couture and Pierre [26], Ingraham et al. [27]. implemented the true triaxial compressive test on the high porosity sandstone, and observed strain localization.

In the aspect of meso fracture characteristic, Fan et al. [28] conducted true triaxial creep test on granite to investigate the impact of ordered mica alignment under different lateral stress directions. Theoretical explanations for the differences in mesoscopic fracturing process of granite with different mica orientations were revealed. In the aspect of micro fracture characteristic, Ma and Haimson investigated the development features of micro cracks in sandstone under true triaxial stress [29,30]. Gao et al. investigated the micro fracture characteristics of marble under the low *σ*_3_ level (*σ*_3_ ≤ 10 MPa), and noticed that the fracture surface was dominated by the tensile patterns [31]. Li et al. analyzed the micro fracture characteristics of the sandstone under the high *σ*_3_ level (*σ*_3_ = 20 MPa), and observed that the fracture surface was dominated by the shear patterns [32]. The analyses of the rock failure under true triaxial stress in above research are mostly limited to a single scale, and the correlation between the macro failure mode and the micro fracture characteristic has not yet been established. More importantly, most of the existing research has focused on the failure of the intact rock, the investigation on the influences of the joint or the flaw on the fracture characteristics of the rock under the true triaxial stress is still in the preliminary exploration stage.

Chang et al. carried out the research on the failure mode of the sandstone with a single pre-existing flaw under the true triaxial stress, and analyzed the macro failure features [33]. However, the types of the cracks initiated from the pre-existing flaw were not summarized, and the failure mechanism was not elucidated. Gao et al. conducted true triaxial fracture test on the marble with a natural stiff joint, and found the geometrical relationship between the directions of the joint normal and the *σ*_1_ was the key factor controlling the failure mode of the rock [34,35]. Whereas, the failure mechanism was also not fully explored. The small number of the existing research has confirmed the significant impact of the flaw on the fracture of the rock, however, the failure mechanisms of the rock containing a joint or flaw under the true triaxial stress is not yet revealed. Therefore, there is an urgent need to conduct the true triaxial fracture test on the flawed rock.

In this research, the intact and pre-flawed sandstones which contains a single pre-existing flaw are adopted to conduct the true triaxial fracture test. By analyzing the macro failure mode, 7 types of basic cracks are summarized. Based on the multi-scale fracture characteristic, the fracture mechanisms of the basic crack are identified. On the basis of the quantity statistics of the cracks with different mechanisms, the influences of the true triaxial stress state and the pre-existing flaw on the rock failure mechanism are revealed. Compared to the existing research, the novelty of this work can be summarized as follows: ⅰ) The basic types of cracks composing the failure of the rocks are summarized; ii) It is found that the fracture characteristics have a good consistency at different scales, and the fracture mechanisms of the basic cracks are judged; iii) The influence of the true triaxial stress state and the pre-existing flaw on the failure mechanisms of the rocks are analysed. The relevant research results have a certain guiding effect on the stability of the surrounding rock and the disaster prevention in deep underground engineering.

## 2. Experimental methodology and technical route

### 2.1. Experimental condition

**Fig 1:**
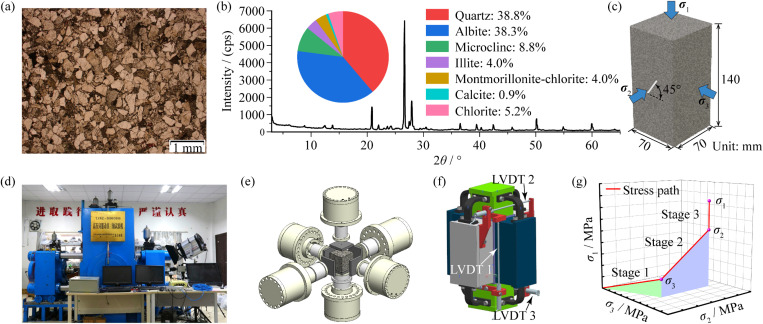
Sandstone and true triaxial apparatus: (a) grain structure and (b) mineral composition of sandstone, (c) sizes and structure of specimen, (d) rigid loading type rock true triaxial apparatus and (e) its loading structure, (f) rigid platen and strain measurement, (g) loading path.

To ensure experimental reliability, sandstone obtained from a quarry in Sichuan Province of China is adopted to manufacture the specimen. The uniaxial compressive strength of the sandstone is 88.48 MPa, and the elasticity modulus and the poisson’s ratio are 14.08 GPa and 0.20, respectively. On the microscopic scale, the sandstone presents a medium-fine grained structure. The microplariscope results indicate that the size of the mineral grains mainly ranges from 0.06 ~ 0.5 mm, of which the grain size is concentrated in the range of 0.25–0.5mm and the porosity is about 6.60%, as shown in Fig 1a. In addition, according to the X-ray fluorescence (XRF) results, the main mineral components include: quartz (38.8%), albite (38.3%), microcline (8.8%), montmorillonite-chlorite (4.0%), illite (4.0%), and calcite (0.9%), chlorite (5.2%), as shown in Fig 1b.

To minimize the effects of material anisotropy and heterogeneity on the test results, the sandstone utilized for experiments is taken from a rock block along the same cutting direction. The P-wave velocities detected along three orthogonal directions are very close, with a difference of less than 5% (the average wave velocity in the sandstone is 2960 m/s).

To study the influence of the flaw on the rock failure, the experiment includes intact specimens and specimens containing a single pre-existing flaw, of which the size is 70 mm × 70 mm × 140 mm. For the pre-flawed specimen, a run through flaw of which the dip angle is 45° is setting in the middle of the *σ*_2_ loading face, as shown in Fig 1c. The length of the pre-existing flaw (2*a*) is 20 mm, and the thickness is 1.5 mm.

In the true triaxial tests, the intermediate and minimum principal stresses (*σ*_2_ and *σ*_3_) have significant influences on the fracture behaviour of rocks. In this study, the variation of stress states is considered, and 12 testing conditions are carried out. To study the influence of *σ*_3_, the value of *σ*_2_ is fixed at 30 MPa, and *σ*_3_ is set to 2 MPa, 15 MPa and 30 MPa. In addition, the influence of *σ*_2_ is examined, in which *σ*_3_ is fixed at 30 MPa and *σ*_2_ is set to 50 MPa, 100 MPa and 150 MPa, as shown in Table 1. All the tests were performed at room temperature of 25 °C and 70% relative humidity

**Table 1:** Specimen type and stress state setting in each condition (Unit: MPa).

Type	Serial number	Type	Serial number	*σ*_2_	*σ*_3_
Intact rock	I-1	Pre-flawed rock	A45-1	30	2
	I-2		A45-2	30	15
	I-3		A45-3	30	30
	I-4		A45-4	50	30
	I-5		A45-5	100	30
	I-6		A45-6	150	30

### 2.2. Experimental apparatus

The experiment is carried out by the true triaxial testing machine developed by Guangxi University (Fig 1d). The stiffness of the machine exceeds 9 GN/m, which guarantees the total stress-strain curves in 3 loading direction can be obtained. This machine is a rigid-plate type (Fig 1e), of which the loading capacities are 5000 kN in *σ*_1_ and *σ*_2_ directions and 3000 kN in *σ*_2_ direction. During test, the specimen is sealed by six rigid platens. To avoid the interference between rigid loading platens, a set of overlapping platens was used in this experiment (Fig 1f). The strains of the specimens are monitored by 3 LVDTs.

Fig 1g shows the loading path of the true triaxial test in this research, which can be divided into three sequential stages:

i) The loading stage of hydrostatic pressure, in which the same principal stresses (*σ*_1_ = *σ*_2_ = *σ*_3_) are applied to all specimen surfaces with a loading rate of 0.5 MPa/s until the stresses reach the design value of *σ*_3_;
ii) The loading stage of intermediate principal stress, in which *σ*_3_ is maintained at the design value, and *σ*_1_ and *σ*_2_ increase simultaneously to the design value of *σ*_2_ with a rate of 0.5 MPa/s;
iii) The loading stage of maximum principal stress, in which *σ*_2_ and *σ*_3_ remain constant, while *σ*_1_ increases at a rate of 0.5 MPa/s within the elastic deformation stage of the specimen. After the *ε*_3_ vs. *σ*_1_ curve bends, the stress-control loading mode of *σ*_1_ is converted to the strain-control mode with a rate of 6 × 10^-4^/min, and the continuous increase in *σ*_1_ causes specimen failure.

### 2.3. Technical route

**Fig 2:**
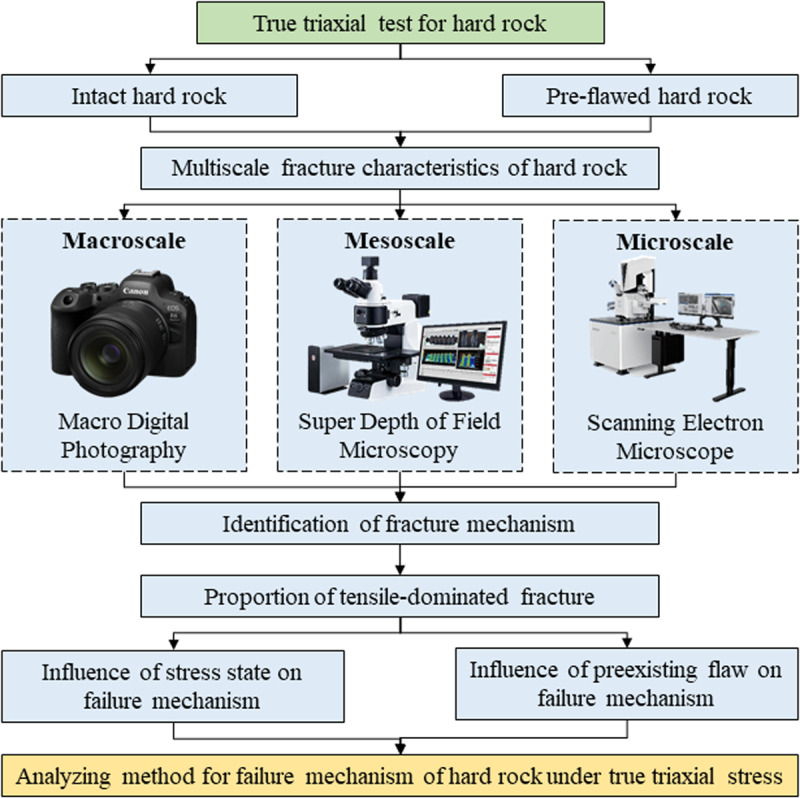
Technical route of this research.

The technical route of this research is shown in Fig 2. By true triaxial test, the multiscale fracture characteristics of the hard rock are studied. The methods of macro photography, microscope, and scanning electron microscope (SEM) are adopted to identify the macroscale, mesoscale, and microscale fracture characteristics, respectively. Then, the fracture mechanism of the basic fracture is identified. Based on the statistics of the quantities of the basic fractures, the proportion of tensile-dominated fracture are calculated, and the influences of the stress state and the pre-existing flaw on the failure mechanism of the hard rock are revealed.

## 3. Macroscopic failure mode

In this section, the macroscopic failure modes of the intact and pre-flawed rocks are presented, and the basic cracks composing the failure of the rock are summarized. Based on the statistic of the crack quantity in each condition, the influence of the pre-existing flaw on the failure mode of the rock is analyzed.

**Fig 3:**
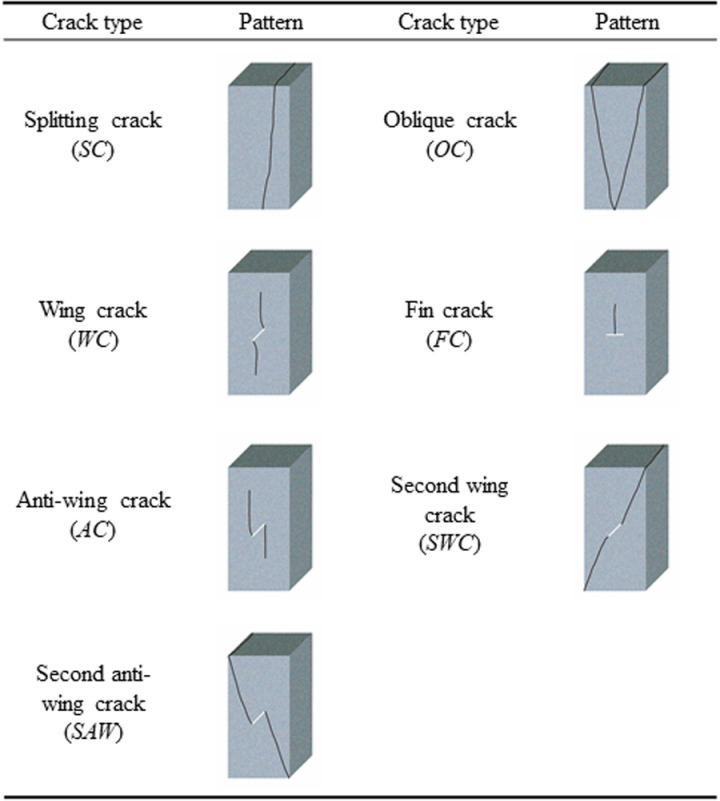
Basic crack type in true triaxial test [ 36–38].

**Fig 4:**
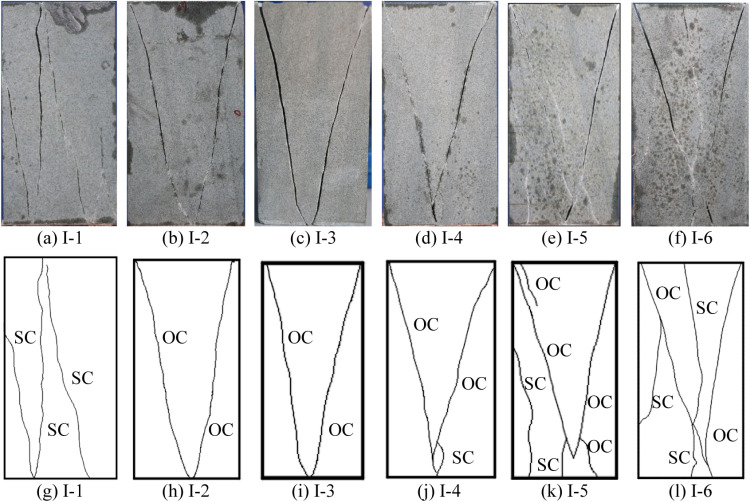
Failure modes of intact rock.

**Fig 5:**
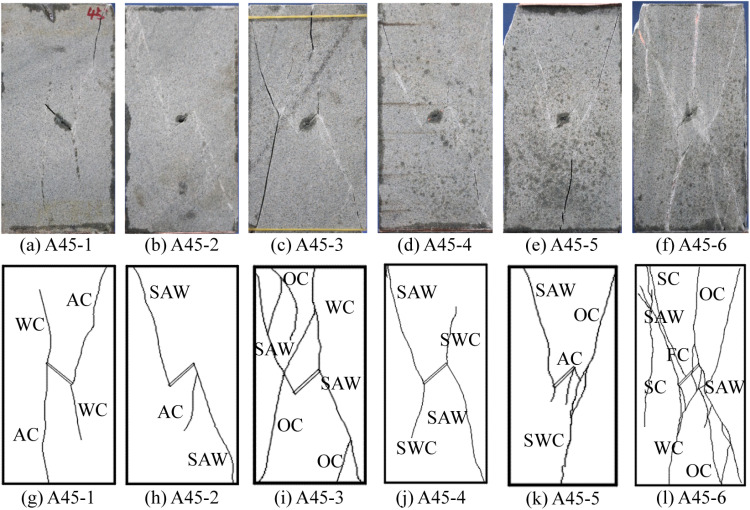
Failure modes of pre-flawed rock.

By analyzing the macro fracture features and patterns, 7 types of basic cracks are identified, as shown in Figs 3–5 are the macro failure modes of the intact rocks and the pre-flawed rocks. These basic cracks appeared in the previous literatures [36–38], so the names of the basic cracks follow those in the previous literatures. However, in the previous literatures, it is rare to observe all 7 types of basic cracks in one test. That is duo to the test in the previous literature is mostly conducted under the uniaxial or conventional triaxial stress, which neglects the effect of the intermediate principal stress on the failure of the rock. In this work, the factors influencing the initiation and development of the crack include the minimum principal stress, the intermediate principal stress, and the pre-existing flaw, which is more complex than those in the previous literatures. Therefore, the types of the cracks in this work are more abundant.

The macro failure modes of the rock are controlled by the true triaxial stress and the pre-existing flaw. For the intact rock, the failure is resulted from the splitting crack and the oblique crack. Maintaining *σ*_2_ at 30 MPa, when the value of *σ*_3_ is low (I-1 condition, *σ*_3_ = 2MPa), the failure of the rock is caused by 3 splitting cracks (Fig 4a and g). When *σ*_3_ is raised to 15 MPa and 30 MPa, the failure of the rock is gradually dominated by oblique cracks which coalesce into a V shape (Fig 4b, c, h and i). When *σ*_3_ is maintained constant, with *σ*_2_ increasing, although the failure of the rock is still dominated by the oblique crack, the splitting crack reappears and its quantity increases. For instance, when *σ*_2_ increases to 150 MPa, the quantity of the splitting crack reaches 3. These splitting cracks joint and coalesce with oblique cracks, which induces a higher broken degree of the rock, as shown in Fig 4f and l.

For the pre-flawed rock, the constitution of the fracture becomes more complex. Maintaining *σ*_2_ constant, in the A45-1 condition (*σ*_3_ = 2 MPa), the growth of the wing crack is limited, and the failure of the rock results from the propagations of the anti-wing crack (Fig 5a and g). In the A45-2 condition (*σ*_3_ = 15 MPa), the wing crack disappears and the propagation of the ant-wing crack is limited. The failure of the rock is induced by the secondary anti-wing crack (Fig 5b and h). When *σ*_3_ increases to 30 MPa, the ant-wing crack disappears, the failure of the rock is induced by the coalescence of several oblique cracks, secondary wing cracks, and secondary anti-wing crack (Fig 5c and i). The disappearances of the wing crack and the anti-wing crack with the increasing *σ*_3_ were also reported by Yang et al. [39].

When maintaining *σ*_3_ at 30 MPa, with the increase of *σ*_2_, the quantities of splitting crack, wing crack, and anti-wing crack increase. Under the A45-5 condition (*σ*_2_ = 100 MPa), anti-wing crack reappears. When *σ*_2_ increases to 150 MPa, there are as many as 5 types of crack appear after the failure of the rock, which results in a very high broken degree of the rock, as shown in Fig 5f and l. The reappearances of the wing crack and the anti-wing crack with the increasing *σ*_2_ were also observed by Chang et al. [33]. The reasons underlying this phenomenon are analyzed in section 4.3.

**Fig 6:**
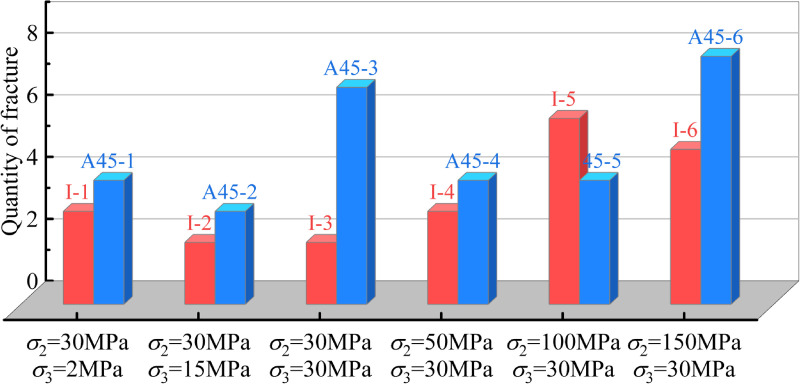
Comparison of crack quantity between intact and pre-flawed rock.

Comparing to the intact rock, the failure of the pre-flawed rock is mainly induced by the development of the new cracks initiated from the pre-existing flaw, with more crack types and more complex failure modes. It is deduced that the pre-existing flaw has a nonnegligible impact on the rock failure mode. Therefore, the quantities of each type of the basic crack are counted, as shown in Table 2, and the total quantities of the intact and pre-flawed rocks under the same stress state are compared (Fig 6)

**Table 2:** Statistic of crack quantity of rock in each condition.

Condition	WC	FC	SC	AC	SWC	SAW	OC	N	Nt	Ns	Pt/ %
I-1	/	/	3	/	/	/	/	3	3	/	100
I-2	/	/	/	/	/	/	2	2	/	2	0
I-3	/	/	/	/	/	/	2	2	/	2	0
I-4	/	/	1	/	/	/	2	3	1	2	33
I-5	/	/	2	/	/	/	4	6	2	4	33
I-6	/	/	3	/	/	/	2	5	3	2	60
A45-1	2	/	/	2	/	/	/	4	4	/	100
A45-2	/	/	/	1	/	2	/	3	1	2	33
A45-3	1	/	/	/	/	2	4	7	1	6	14
A45-4	/	/	/	/	2	2	/	4	0	4	0
A45-5	/	/	/	1	1	1	1	4	1	3	25
A45-6	1	1	2	/	/	2	2	8	4	4	50

In Fig 6, except for the *σ*_2_ = 100 MPa, *σ*_3_ = 30 MPa condition, in other conditions, the crack quantities in the pre-flawed rocks are always more than those in the intact rock. Combining the phenomenon that most of the cracks initiate from the pre-existing flaw, it can be concluded that pre-existing flaw has a significant promoting effect on the crack initiation and development, and contributing to the rock failure. This promoting effect is associated with the high stress concentration in the tips of the pre-existing flaw. Once the stress concentration exceeds the fracture toughness, the crack initiates, which in turn further strengthens the stress concentration, and contributes to the formation of more cracks.

## 4. Failure mechanism of rock

### 4.1. Multi-scale fracture characteristic

The characteristics of the crack surface are the directly records of the failure process of the rock. In this research, the multi-scale fracture characteristics of the crack surfaces are analyzed to identify the fracture mechanisms of the basic cracks. The influences of the stress state and the pre-existing flaw on the failure mechanisms of the rock are elaborated. Taking the splitting crack (I-1 condition) and the oblique crack (I-2 condition) as examples to expound the analysis process.

**Fig 7:**
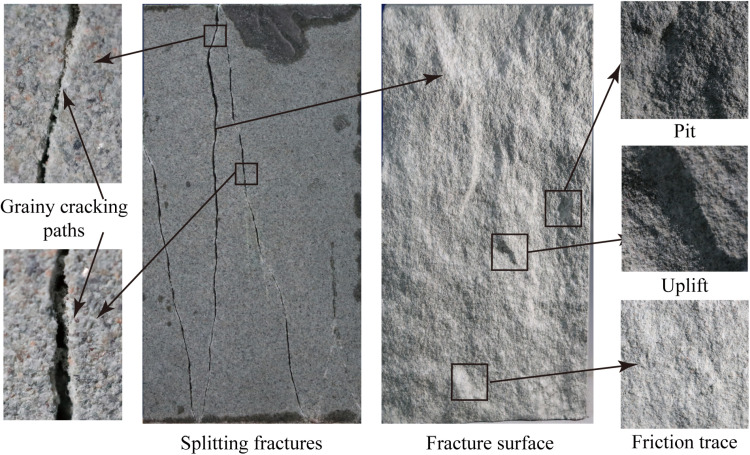
Macroscopic pattern and fracture characteristic of splitting crack.

Fig 7 is the macro fracture characteristics of the splitting crack. The cracking path presents a strong sense of graininess. The crack surface is rough with a large degree of waviness, and bulges and pits can be observed. There are also a few sporadic friction traces on the crack surface. Overall, the crack surface is clean, and only a small amount of rock powder and debris accumulate in the friction regions. These rough but clean features consistent with those of the tensile fracture of sandstone in Brazilian disc split test [40,41].

**Fig 8:**
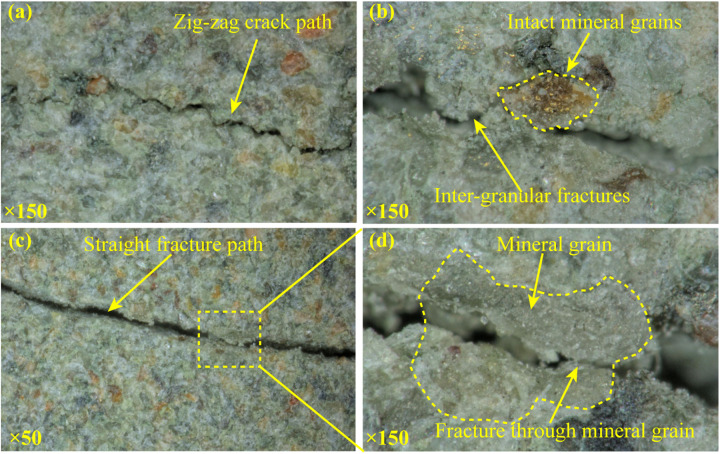
Mesoscopic fracture characteristics of splitting crack.

Fig 8 is the mesoscopic fracture characteristics. Micro cracks propagate along the grain boundaries forming a zigzag path (Fig 8a). This is because the tensile strength of the clay between the sandstone grains is much lower than that of the grain. Under the tensile effect, the clay will be broken, and the crack propagates along the boundary. This inference can be verified by the study conducted by Yang et al. [42].

Only a few cracking paths are flat and straight (Fig 8c), and run through the grains (Fig 8d). This is due to that under the shear effect, the crack develops along the direction of the shear stress, crushing the grains and forming a straight path.

**Fig 9:**
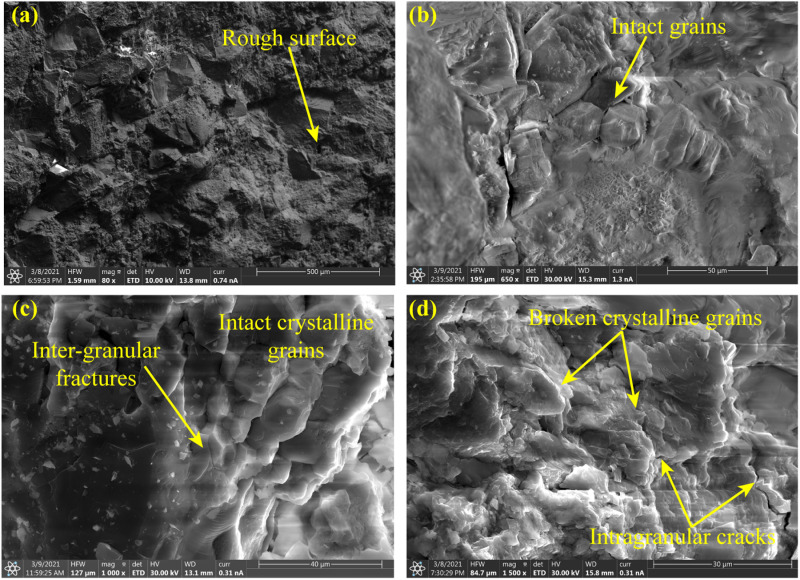
Microscopic characteristics of splitting fracture surface.

Fig 9 is the microscopic fracture characteristics of the splitting crack. The crack surface presents an obvious rough characteristic (Fig 9a). The grains on the surface remain intact, and their profiles are clear (Fig 9b). It is interesting that the rough but clean fracture surface highly consistent on the macro and micro scales. Microcracks develop along the boundaries of the crystalline grains, and intergranular fracture is the major fracture mode (Fig 9c). This is also reported by Zhang et al. [43] that the crystalline grains on the tensile fracture surface of raw coal remain intact. All of the above are typical characteristics of tension fracture. At the same time, there are also a small number of broken crystalline grains in some regions, and intragranular cracks are observed, which resulted from shear fracture (Fig 9d).

**Fig 10:**
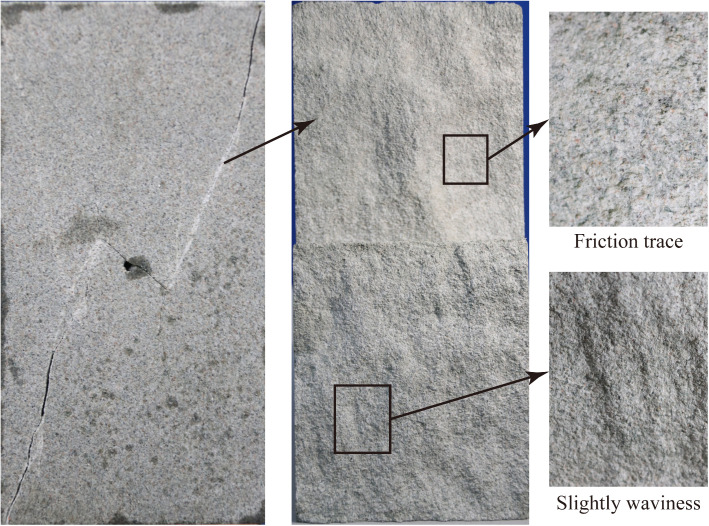
Macroscopic pattern and fracture characteristic of oblique crack.

Fig 10 is the macro fracture characteristics of the oblique crack. The cracking path is straight, and the crack surface is flat, with no obvious waviness. The fraction traces are widely spread, and a large amount of the rock powder and debris adhere. The flat surface and the fraction traces are similar to those of the fracture of the sandstone in the shear test reported by Zhao et al. [44].

**Fig 11:**
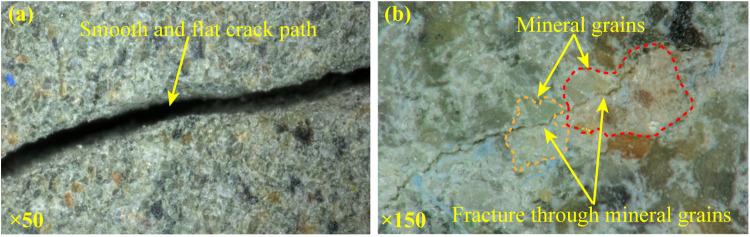
Mesoscopic fracture characteristics of oblique crack.

Fig 11 shows the mesoscopic cracking characteristics of oblique crack. The cracking path still exhibits straight characteristics (Fig 11a). This is because the undulations of the cracking path have been abraded by shearing effect. This is also confirmed by many crack paths crossing the mineral grains (Fig 11b). The straight cracking path is also observed by Yang et al. [42].

**Fig 12:**
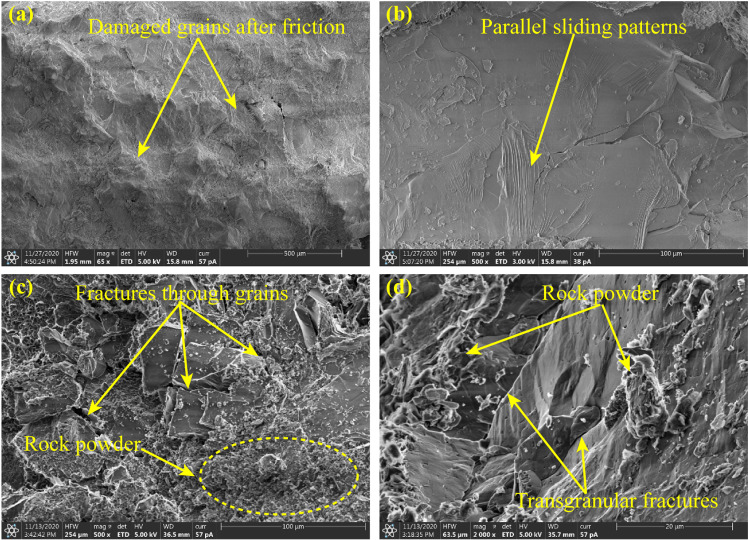
Microscopic fracture characteristics of oblique crack.

Fig 12 is the SEM results of the oblique fracture surface. On the surface of the oblique fracture, most of the mineral grains are abraded and damaged, the graininess of the surface is much weaker than that of vertical splitting fracture (Fig 9a and Fig 12a). The parallel sliding patterns are widespread on the fracture surface (Fig 12b), and micro fractures running through the mineral grains are detected (Fig 12c). When the image is amplified to crystal size, the trans-granular fractures of the crystal mineral such as quartz can be observed (Fig 12d). These microscale fracture characteristics are also observed in the shear fracture reported by Li et al. [45] and Zhao et al. [46]. All of the above are typical characteristics of the shear fracture. Hence, the oblique fracture is identified as shear fracture.

### 4.2. Identification of fracture mechanism

Combining the observed multi-scale fracture characteristics and the literature supports, it can be concluded that: the tensile fracture presents a rough but clean surface with a high degree of fluctuation on the macroscale, a zigzag cracking path propagating along the grain boundaries on the mesoscale, and an inter-granular fracture mode with intact mineral particles or crystalline grains on the microscale; while the shear fracture presents a flat surface with obvious friction traces and a large amount of the rock powder and debris adhere on the macroscale, a smooth and straight cracking path crossing the mineral grains on the mesoscale, and a the trans-granular fracture mode with abraded and damaged mineral particles and crystalline grains on the microscale.

From the above summary, the mechanism of the splitting crack can be identified as mixed-mode fracture dominated by the tensile effect, and the mechanism of the oblique crack can be determined to be shear fracture. using the same method to identify the multi-scale fracture characteristics of the other 5 basic cracks, the fracture mechanisms are identified and divided into four categories, as shown in Table 3.

**Table 3:** Fracture mechanism and classification.

Type	Fracture mechanism	Crack type	Controlling factor
Type Ⅰ	Tension fracture	Wing crack	Only initiating from the pre-existing flaw with a low *σ*_3_ (*σ*_3_ = 2 MPa in this research) or a very high stress difference between *σ*_2_ and *σ*_3_ (*σ*_3_ = 30MPa, *σ*_2_ = 150 MPa in this research).
		Fin crack	
Type Ⅱ	Mixed-mode fracture dominated by tension	Anti-wing crack	Only initiating from the pre-existing flaw, suppressed by *σ*_3_ and promoted by *σ*_2_.
		Splitting crack	Initiation and development independent of the pre-existing flaw, suppressed by *σ*_3_ and promoted by *σ*_2_.
Type Ⅲ	Mixed-mode fracture dominated by shearing	Secondary wing crack	Only initiating from the pre-existing flaw with a high *σ*_3_, less dependent of *σ*_2_.
		Secondary anti-wing crack	Only initiating from the pre-existing flaw with a high *σ*_3_, less dependent of *σ*_2_.
Type Ⅳ	Shear fracture	Oblique crack	Initiation and development independent of the pre-existing flaw, mostly appearing with a high *σ*_3_.

Type I is the tension fracture, including wing crack and fin-shaped crack. Type II is the mixed-mode fracture dominated by the tensile effect, including splitting crack and anti-wing crack. Type III is the mixed-mode fracture dominated by the shear effect, including secondary wing crack and secondary anti-wing crack. Type IV is the shear fracture, including oblique crack.

For the sake of analysis, in Table 3, types I and II are classified as tension-dominated crack, and types Ⅲ and Ⅳ are classified as shear-dominated crack. The total quantity of cracks is denoted as *N*, the quantity of the tension-dominated crack is denoted as *N*_t_, and the ratio of *N*_t_ to *N* is denoted as *P*_t_. Based on the statistical results, the influences of true triaxial stress and pre-existing flaw on the rock failure mechanism are analyzed.

### 4.3. Rock failure mechanism

**Fig 13:**
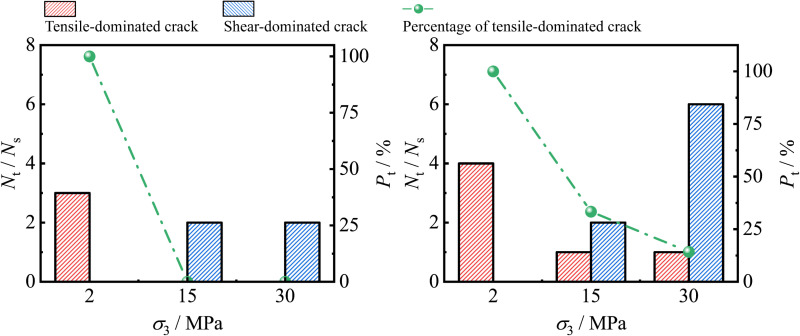
Variation trends of quantities of different crack types and proportions of tensile-dominated cracks with *σ*_3_ increasing.

Fig 13 shows the variation trend of the quantity of different types of cracks and the percentage of tension-dominated cracks as *σ*_3_ increases. Keeping *σ*_2_ constant, when the value of *σ*_3_ is small, the rock failure is dominated by tension-dominated cracks. As *σ*_3_ increases, *P*_t_ decreases, the number of shear-dominated cracks continues to increase, and the rock failure is gradually dominated by shear fractures.

This transition is also qualitatively described by Yang et al. [47] in a conventional triaxial test for sandstone and Gao et al. [35] in a true triaxial test for marble. When the value of *σ*_3_ is small, the constraint on the rock in the direction of *σ*_3_ is weak, and the lateral expansion can be fully developed, promoting the initiation of the tension-dominated crack. As *σ*_3_ increases, the lateral constraint is strengthened, and the initiation of the tension-dominated crack is significantly inhibited. Therefore, the rock failure gradually transforms into compression-shear failure, and the quantity of shear-dominated cracks increases.

**Fig 14:**
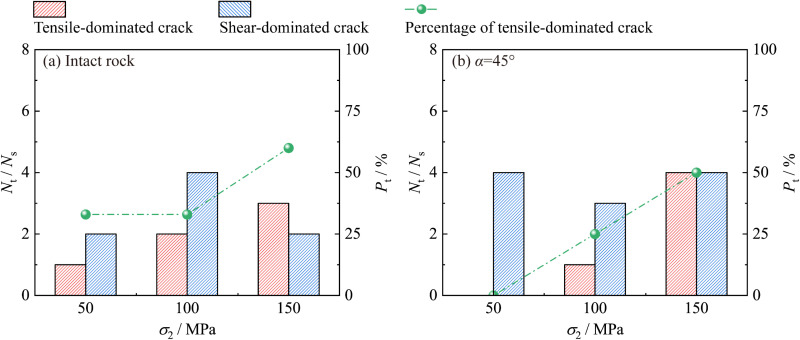
Variation trends of quantities of different crack types and proportion of tensile-dominated cracks with *σ*_2_ increasing.

Fig 14 shows the variation trend of the quantities of different types of cracks and the percentage of tension-dominated cracks as *σ*_2_ increases. When *σ*_3_ remains constant, as *σ*_2_ increases, the quantity and percentage of tension-dominated cracks show an increasing trend. This positive correlation between *σ*_2_ and the tensile fracture is consistent with the findings by Ma and Haimson [29] and Feng et al. [13].

An increase in *σ*_2_ will cause a longer intermediate principal stress loading stage. During this period, the rock is in a triaxial extension stress state (*σ*_1_ = *σ*_2_ > *σ*_3_). When subjected to double extrusion in both the *σ*_1_ and *σ*_2_ directions, the expansion deformation in the *σ*_3_ direction is significantly enhanced, which greatly promotes the initiation of tension-dominated crack. Therefore, the quantity and proportion of tension-dominated crack show an increasing trend.

**Fig 15:**
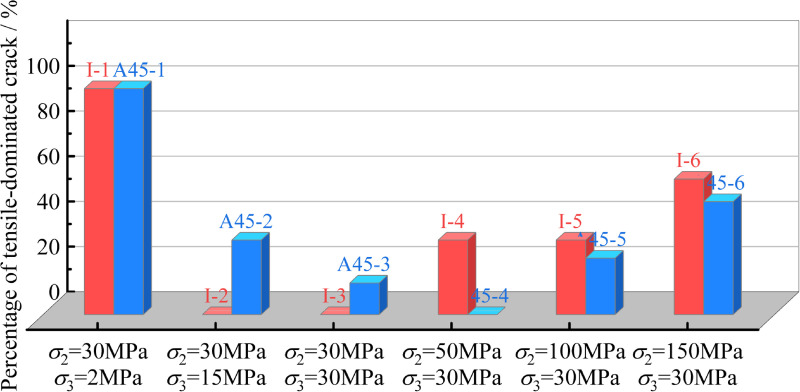
Comparison of percentage of tensile-dominated cracks between intact and pre-flawed rocks under same stress state.

Fig 15 shows the comparison of the percentage of the tension-dominated cracks between the intact and pre-flawed rocks under the same stress conditions. Compared with true triaxial stress, the influence of the pre-existing flaw on the rock failure mechanism is weaker, and the regularity is not obvious. It is deduced that the true triaxial stress will promote the pre-existing flaw closure, enhancing the friction between flaw surfaces, and suppressing the concentration of tensile stress at the flaw tip. Therefore, the true triaxial stress is the decisive factor in controlling the rock failure mechanism, and can effectively weaken the influence of pre-existing flaw.

## 5. Discussion

### 5.1. Influence of the size effect on the fracture characteristics

**Fig 16:**
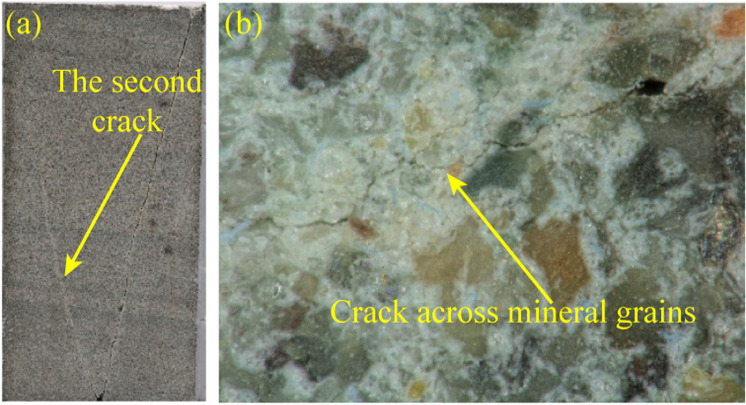
Macroscopic and mesoscopic fracture characteristics of the small specimen: (a) macroscopic fracture characteristics, (b) mesoscopic fracture characteristics.

From the analyses of the splitting crack and the oblique crack in the 4.1 section, the fracture characteristics are in a good consistence on the different scales. One may wonder whether the variation of the specimen size or the size effect would introduce a change to the fracture characteristics. Hence, a small sandstone specimen with a size of 25 × 25 × 50 mm was manufactured to repeat the test with the true triaxial stress state of *σ*_3_ = 15 MPa, *σ*_2_ = 30 MPa. The macroscopic and mesoscopic fracture characteristics of the small specimen are shown in Fig 16.

From Fig 16, it can be observed the macro failure mode of the small specimen is very similar to that of the large specimen in I-2 condition (Fig 4b), which consist of two crack forming a V-shape. If it has to be distinguished, compared to the large specimen, the second crack in the small specimen is not fully developed. The reason may lie in two points: ⅰ) The small specimen has less ability to withdraw the development of the crack. The propagation of the crack is easier to induce the failure of the rock; ⅱ) The boundary effect attract the second crack to propagate to the lateral of the specimen, restricting the development of the crack.

Although the variation of the specimen size brings a slight difference to the failure mode in the macroscopic, the mesoscopic fracture characteristics of the large and small specimen are consistent (Fig 11b and Fig 16b). It can be observed the crack propagates across the mineral grains, which is consistent with that of the oblique crack in the large specimen. Therefore, the size effect has a negligible influence on the multi-scale fracture characteristics. This is because the variation of the size is beyond the order of millimetres. While the mesoscopic and microscopic fracture characteristics are on the order of micrometer and nanometer, respectively. Even the size reduces to 25 mm, it is still much larger than the scales of the mineral grains.

### 5.2. Connecting true triaxial test with engineering application

Connecting the laboratory test with the engineering application is a goal that every geotechnical researcher strives to achieve. In this research, it is found the fracture characteristics are in a good consistence on the different scales. We infer that the fracture characteristics of the rock mass at the engineering scale are also in good a consistence with those at the laboratory scale.

To verify this inference, the spalling failure of the marble in the project of Jinping underground laboratory (CJPL-II) is investigated. CJPL-II is a typical deep high geostress underground engineering of which the burial depth approaches 2400 m, and the maximum and minimum principal stresses exceeds 70 MPa and 30 MPa, respectively [48,49]. After the excavation, the maximum principal stress in the surrounding rock significantly rises, and the minimum principal stress rapidly decreases to nearly 0 MPa, which is very similar to the stress states of I-1 and A45-1 conditions.

**Fig 17:**
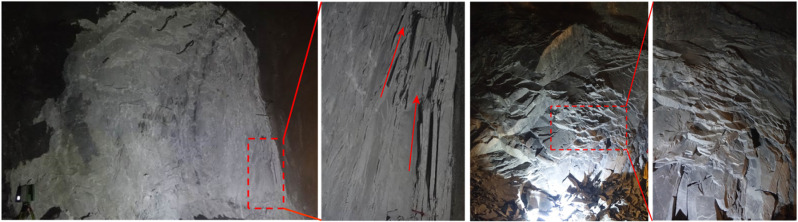
Spalling fracture of surrounding rock in CJPL-II.

The strong loading and unloading effect leads to severe spalling failure in the surrounding rock. Fig 17 is the spalling failure of the surrounding rock in the CJPL-II. The surfaces of the spalling failure have a cross-scale similarity with the fracture surface of the splitting crack, both of which develop vertically parallel to the boundary (lateral side of the specimen or tunnel wall), and present a strong sense of roughness with obvious waviness and unevenness. It is inferred that the formation of the spalling failure is dominated by the tensile fracture which is same as the splitting crack. Therefore, in the deep high geostress hard rock, in order to effectively prevent the spalling failure, the pre-stressed anchor and bolt can be applied to alleviate the unloading effect and restrain the development of the tensile fracture.

## 6. Conclusion

In order to study the rock failure mechanism under general stress state, true triaxial fracture tests are carried out on the intact and pre-flawed rocks. By identifying multi-scale fracture characteristics, the fracture mechanisms of the basic cracks are identified. Based on the statistics of the quantity of cracks, the influences of true triaxial stress and pre-existing flaw on the rock failure mechanism are studied, and the quantitative relationship between the fractal dimension and the fracture mechanism is discussed. The main conclusions are as follows:

(1) Under true triaxial stress, there are seven basic types of cracks after the rock failure, namely splitting cracks, oblique cracks, wing cracks, fin-shaped cracks, anti-wing cracks, secondary wing cracks and secondary anti-wing cracks.
(2) The rock failure mode is controlled by true triaxial stress and pre-existing flaw. By identifying multi-scale fracture characteristics, the fracture mechanism of the basic type of crack is analyzed. Based on the statistics of the quantity of different types of cracks, the influences of true triaxial stress and pre-existing flaw on the rock failure mechanism are analyzed.
(3) As *σ*_3_ increases from 2 MPa to 30 MPa, the percentage of the tension-dominated crack decreases from 100% to zero for the intact rock, and 100% to 14% for the cracked rock. The rock tends to undergo shear failure. With *σ*_2_ increases from 50 MPa to 150 MPa, the percentage of the tension-dominated crack increases from 33% to 50% for the intact rock, and zero to 50% for the cracked rock, which indicates the increase of *σ*_2_ has a significant promoting effect on the development of tensile fracture.
(4) While the pre-existing flaw has a certain promoting effect on the initiation of tensile fracture, the true triaxial stress is the decisive factor in controlling the rock failure mechanism, and can effectively weaken the influence of pre-existing flaw on the rock failure mechanism. It is deduced that the true triaxial stress will promote the closure of the pre-existing flaw closure, enhancing the friction between flaw surfaces, and suppressing the concentration of tensile stress at the flaw tip.
(5) The size effect on the fracture characteristics and the similarity of the fracture surfaces between laboratory and engineering scales are discussed. The size effect has a negligible influence on the multi-scale fracture characteristics, because the variation of the macroscopic size is far beyond the order of the mesoscopic and microscopic fracture characteristics. The surfaces of the spalling failure have a cross-scale similarity with the fracture surface of the splitting crack. It is inferred that the formation of the spalling failure is dominated by the tensile fracture, and the pre-stressed anchor and bolt can be applied to restrain the development of the spalling failure.

## Supporting information

S1 Fig Source file of Fig 4. (PDF)

S2 Fig Source file of Fig 5. (PDF)

S3 Fig Source file of Fig 7. (PDF)

S4 Fig Source file of Fig 8. (PDF)

S5 Fig Source file of Fig 9. (PDF)

S6 Fig Source file of Fig 10. (PDF)

S7 Fig Source file of Fig 11. (PDF)

S8 Fig Source file of Fig 12. (PDF)

## Abbreviations

*σ*_1_: maximum principal stress
SC: splitting crack
*σ*_2_: intermedia principal stress
OC: oblique crack
*σ*_3_: minimum principal stress
WC: wing crack
LVDT: linear variable differential transformer
FC: fin crack
*N*: total quantity of cracks
AC: anti-wing crack
*N*_t_: quantity of the tension-dominated crack
SWC: second wing crack
*P*_t_: ratio of *N*_t_ to *N*
SAW: second anti-wing crack
CJPL-II: project of Jinping underground laboratory.
