# Supplementary figures and images for: A multi-scale observation and crack statistics based method for analyzing failure mechanism of pre-flawed rock under true triaxial stress
Source: PLoS One. 2025 May 23;20(5):e0323809. doi: 10.1371/journal.pone.0323809 (PMC12101716; doi:10.1371/journal.pone.0323809)

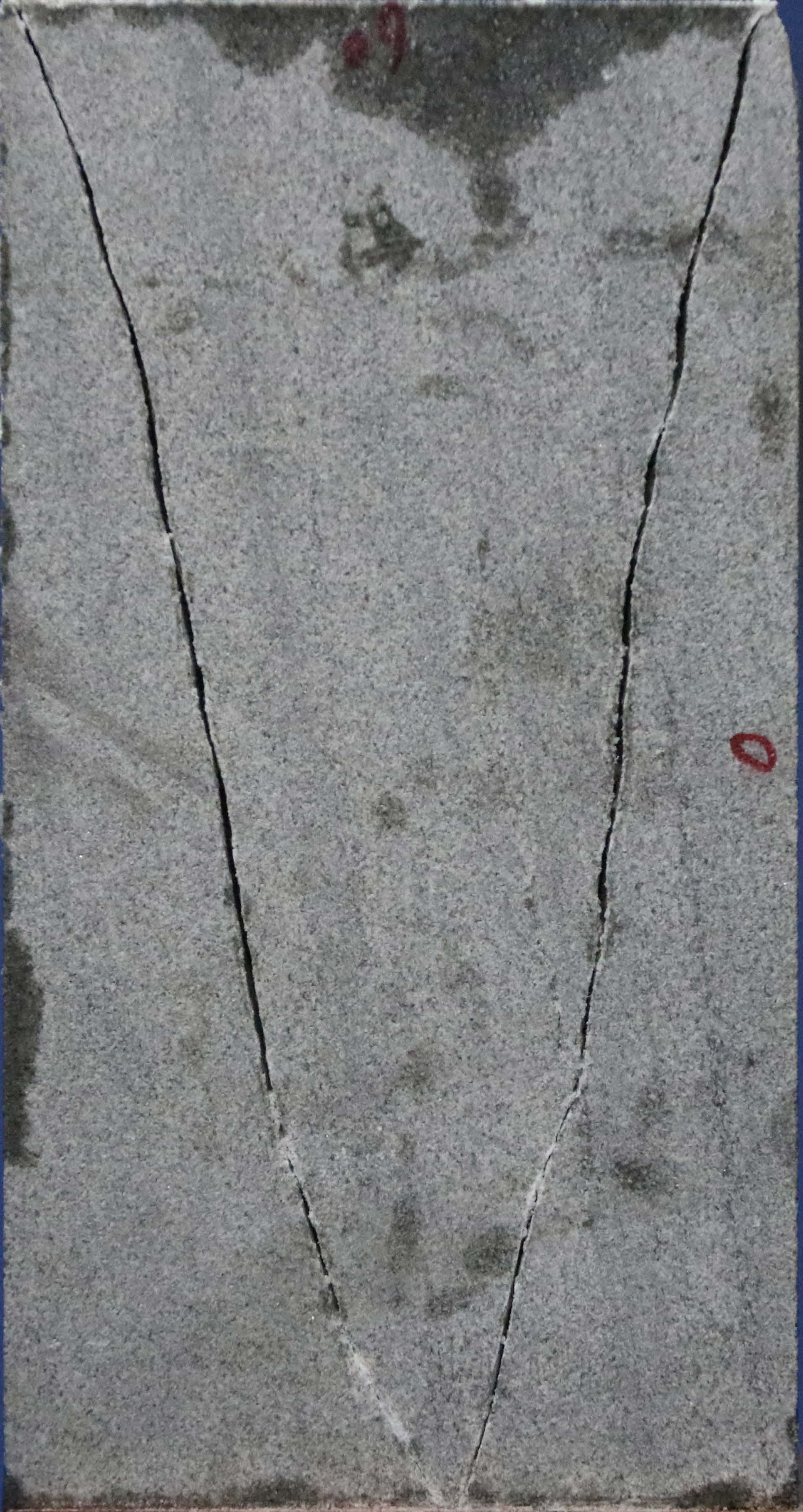

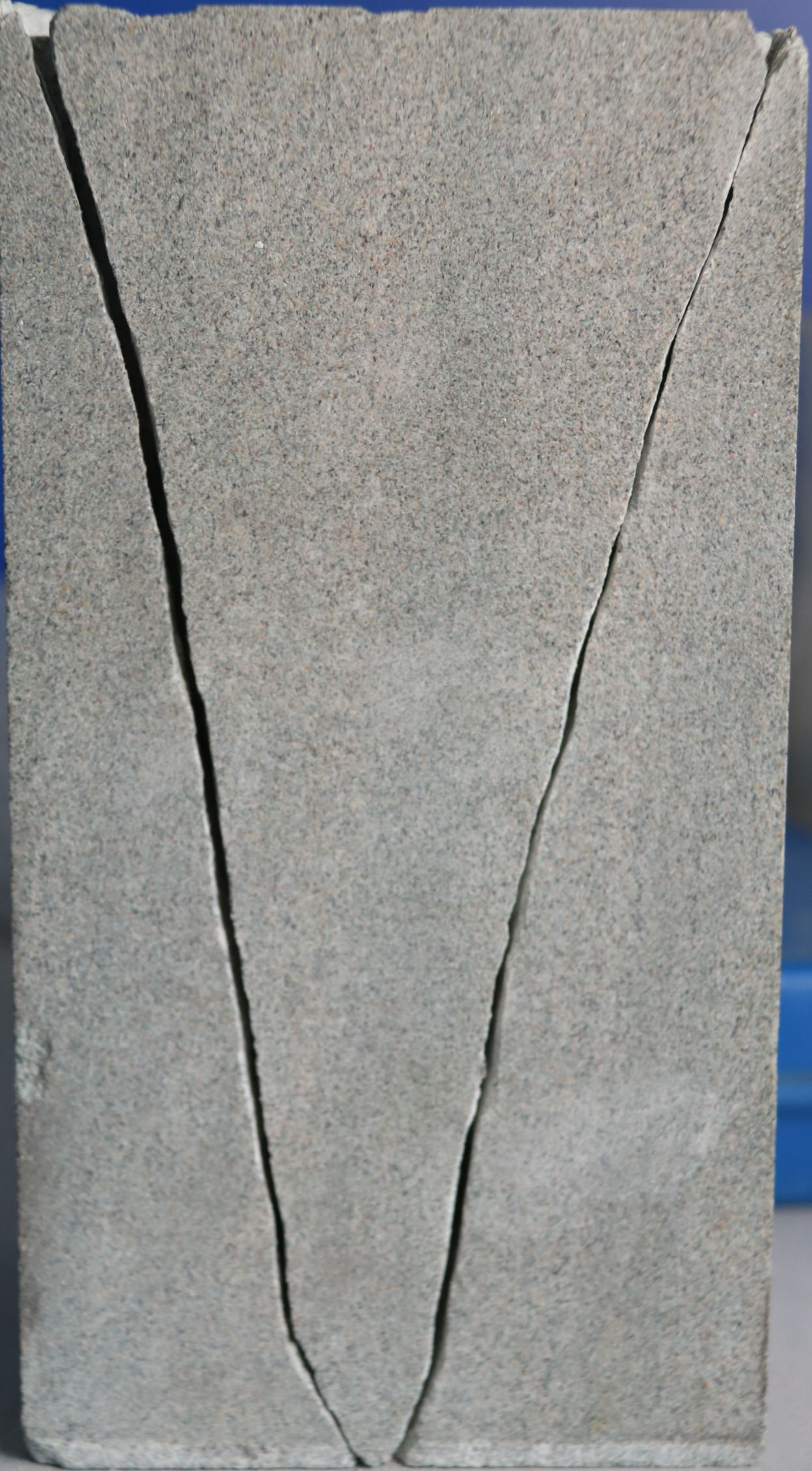

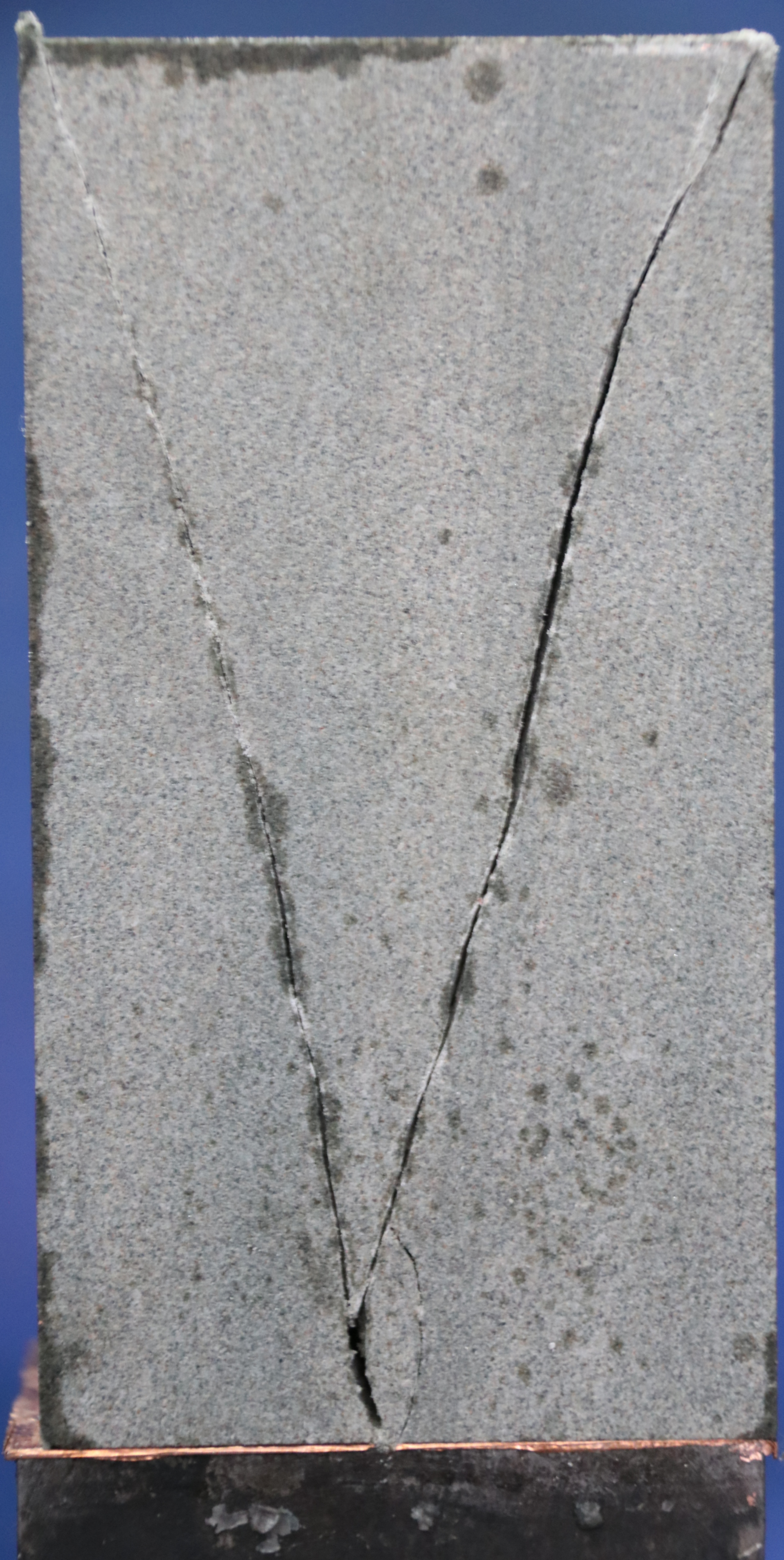

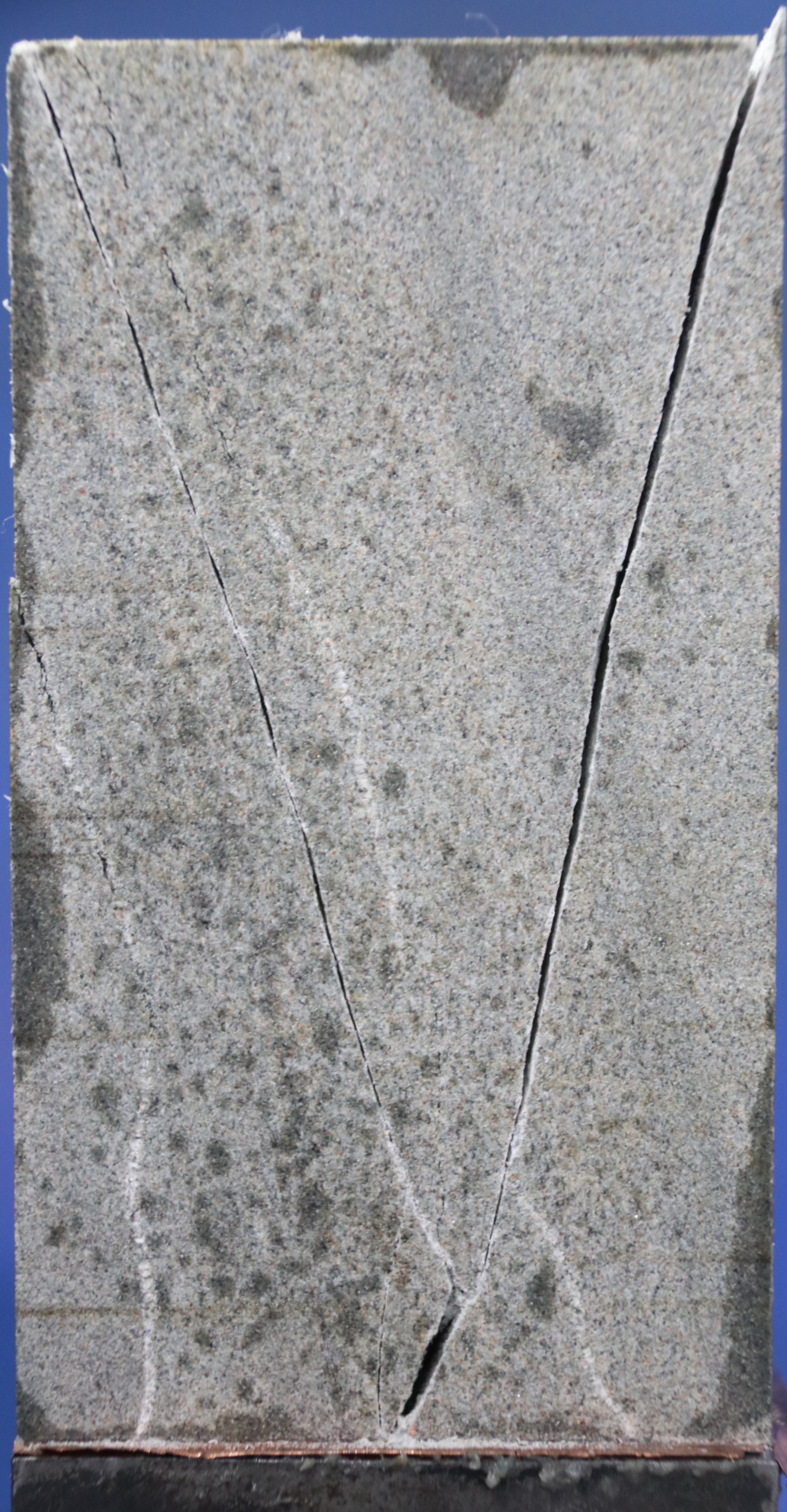

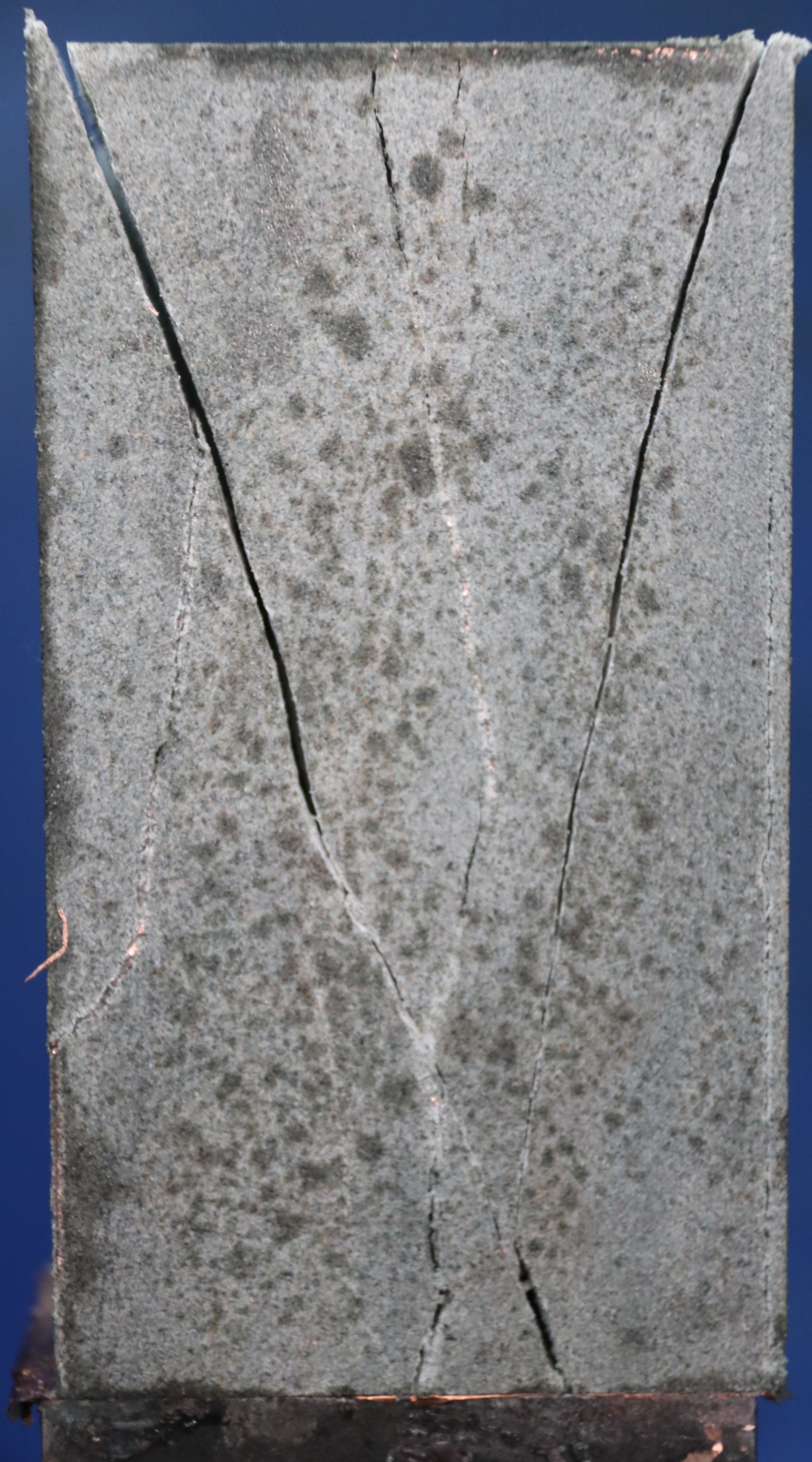

Supplement: S1 Fig — (PDF) [file pone.0323809.s001.pdf]

45

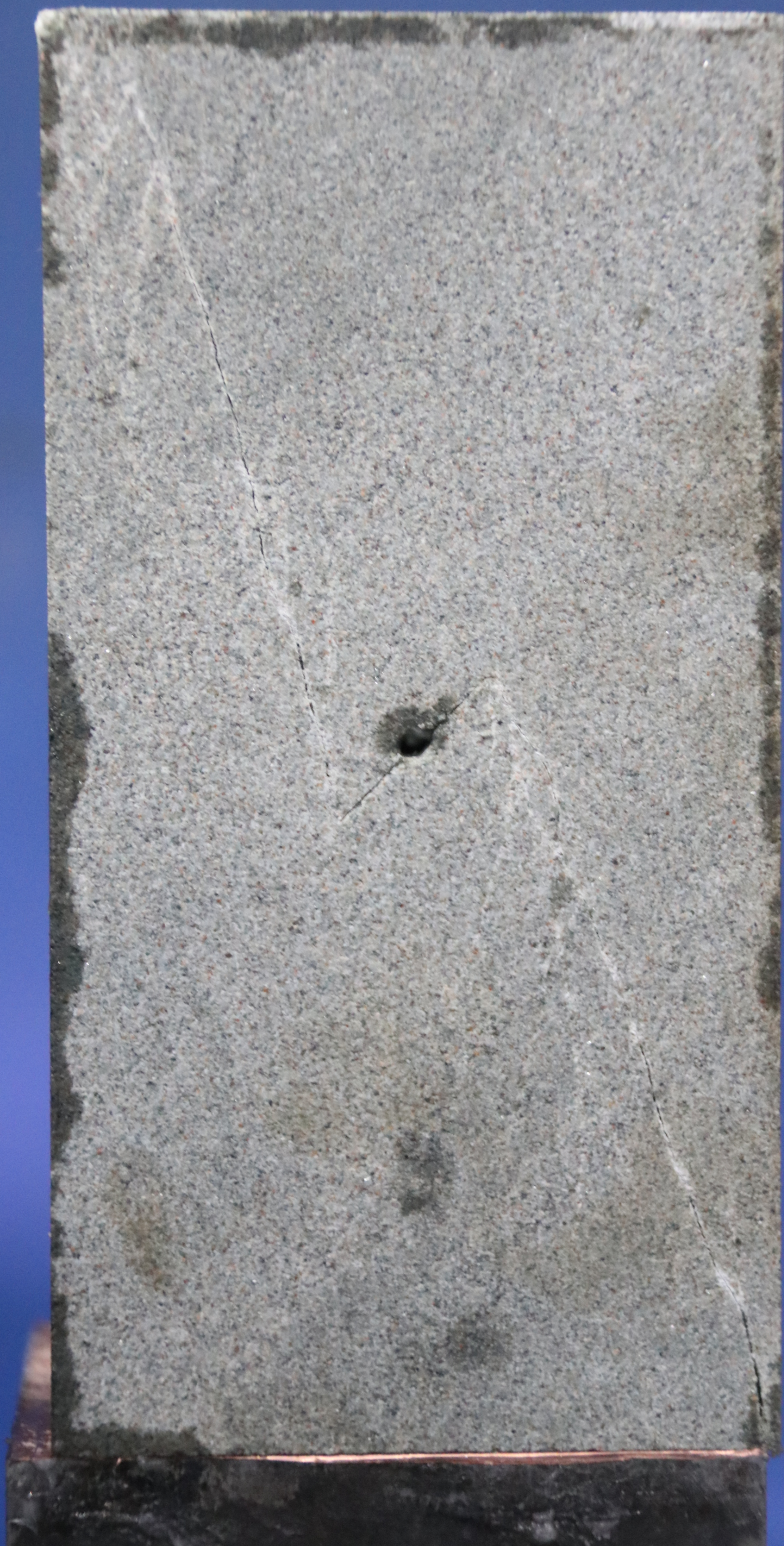

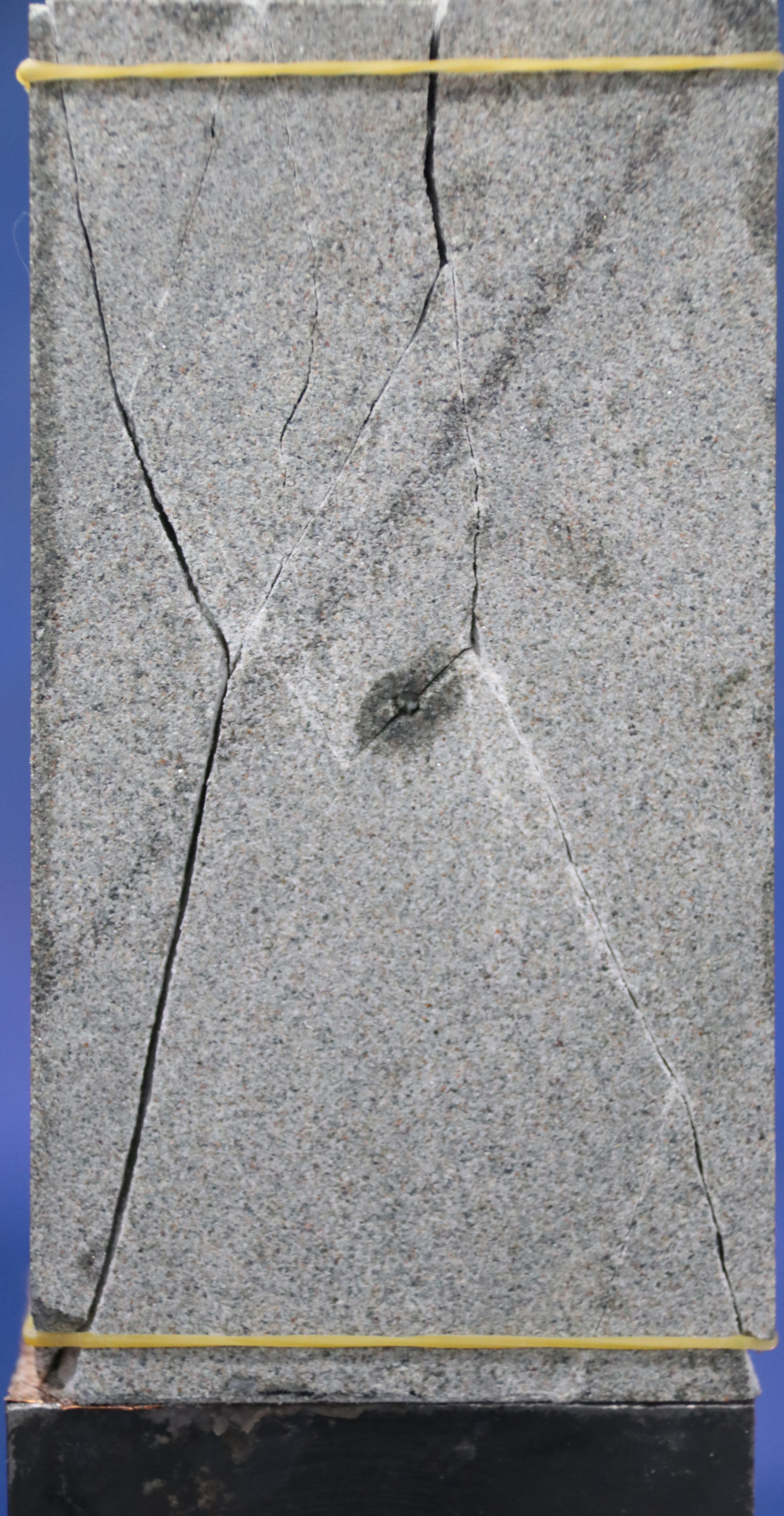

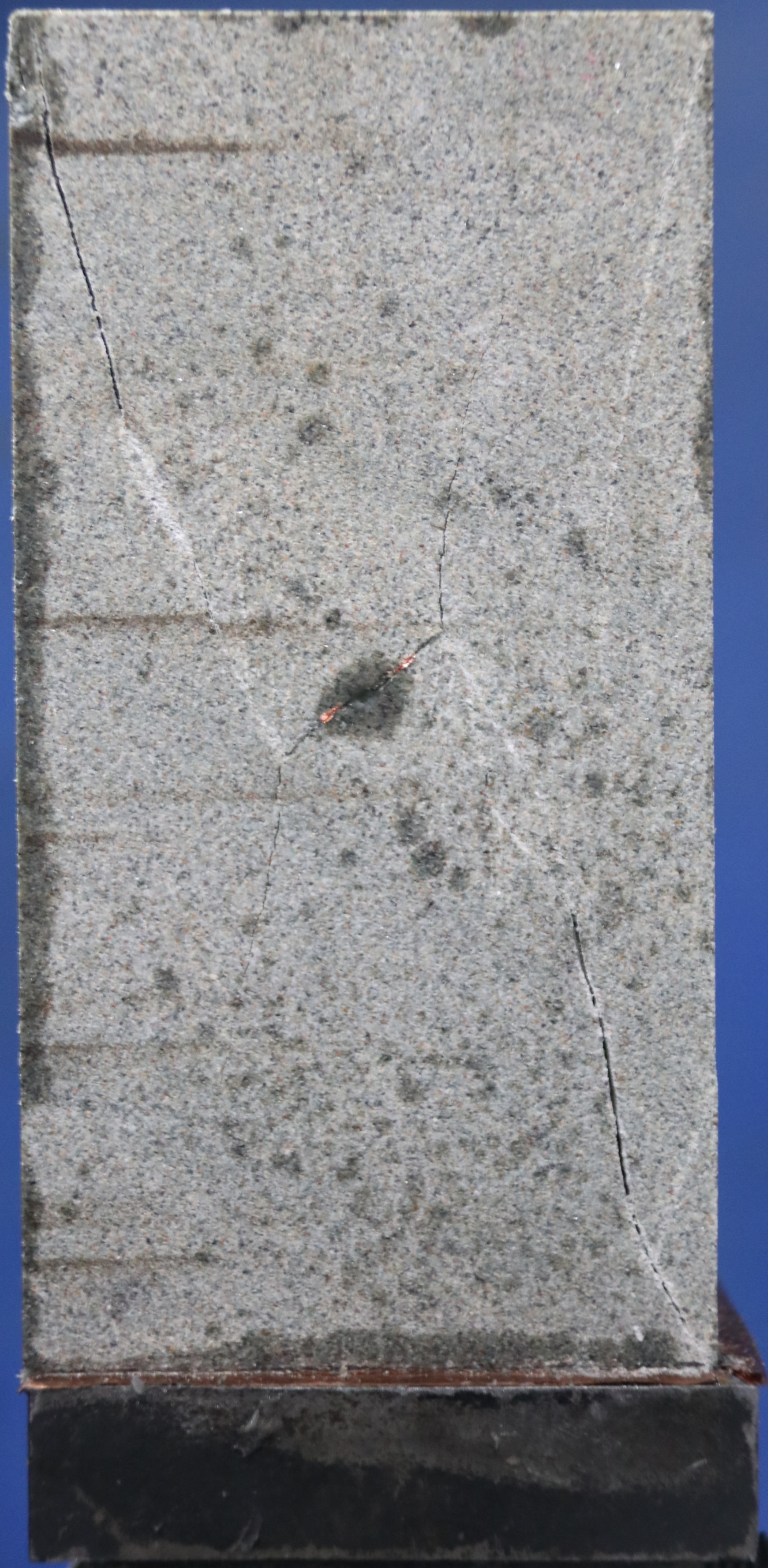

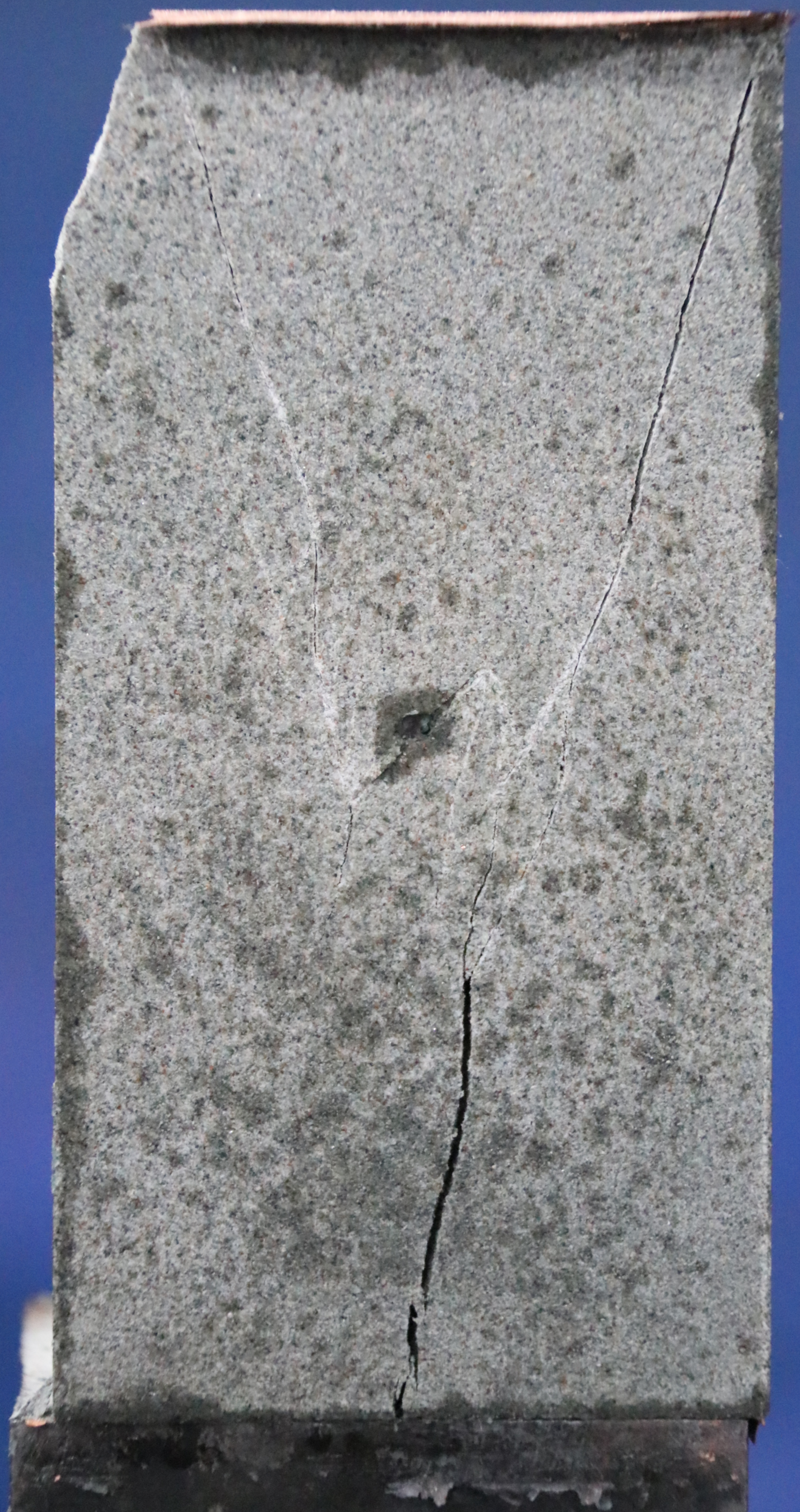

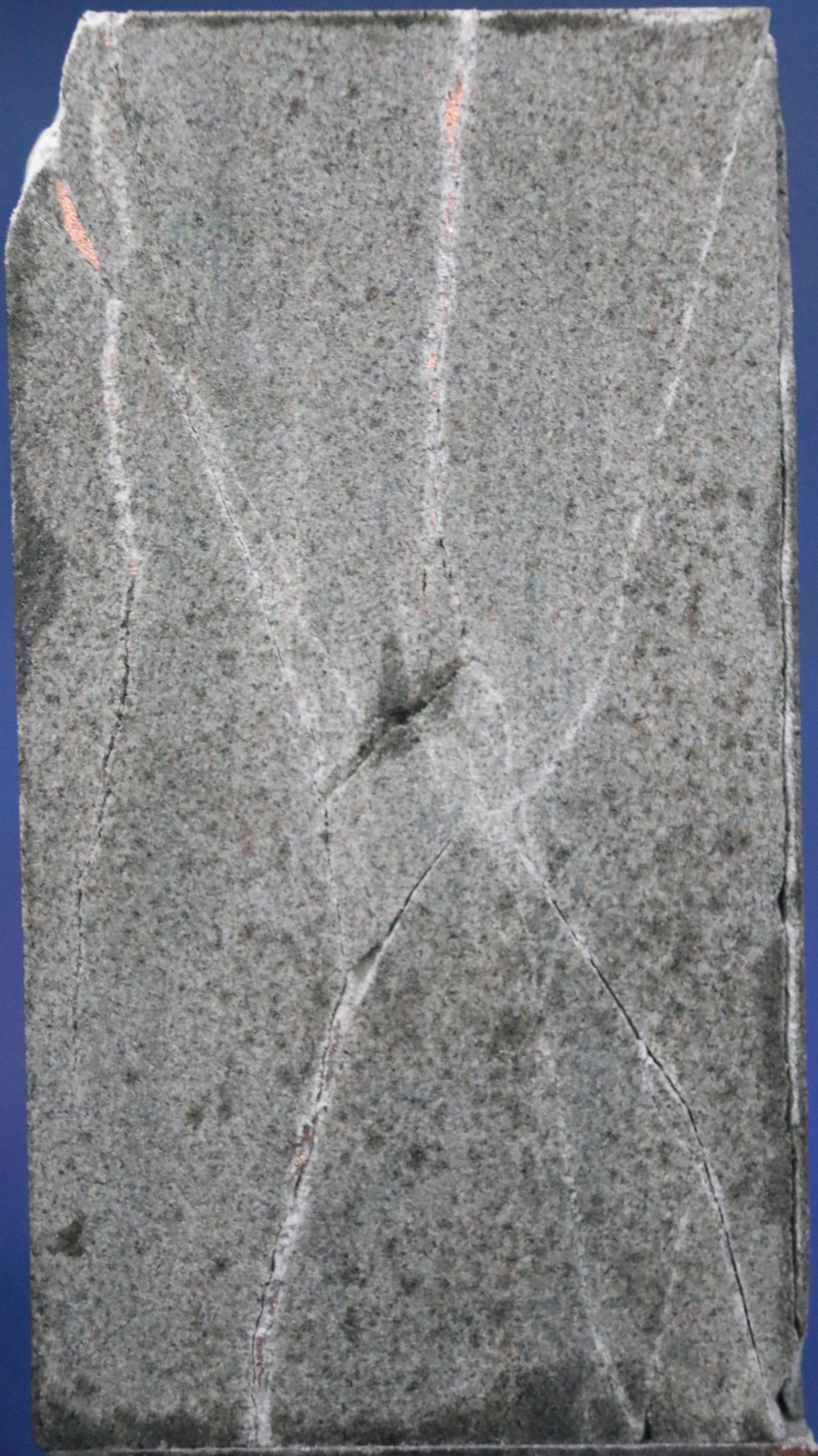

Supplement: S2 Fig — (PDF) [file pone.0323809.s002.pdf]

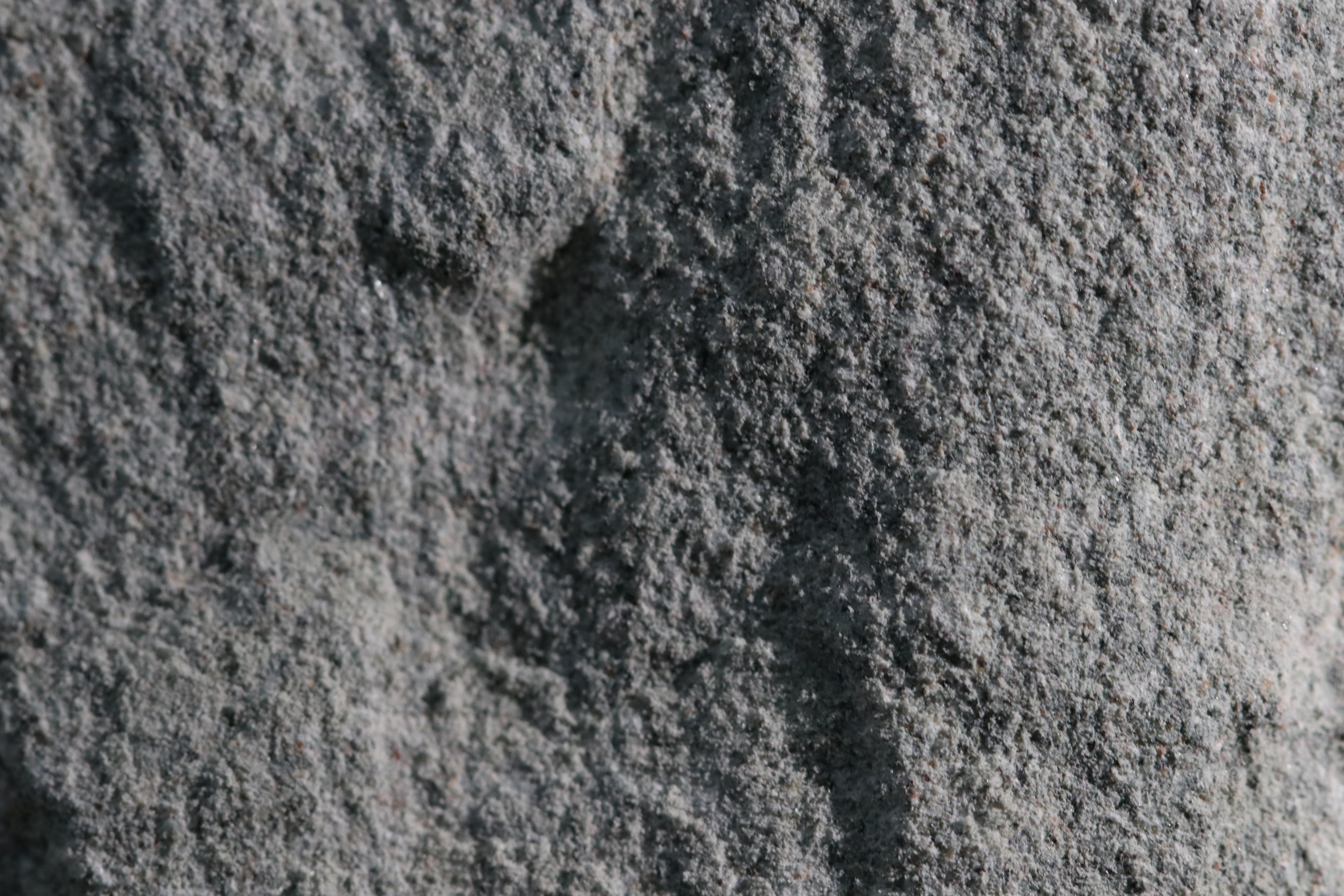

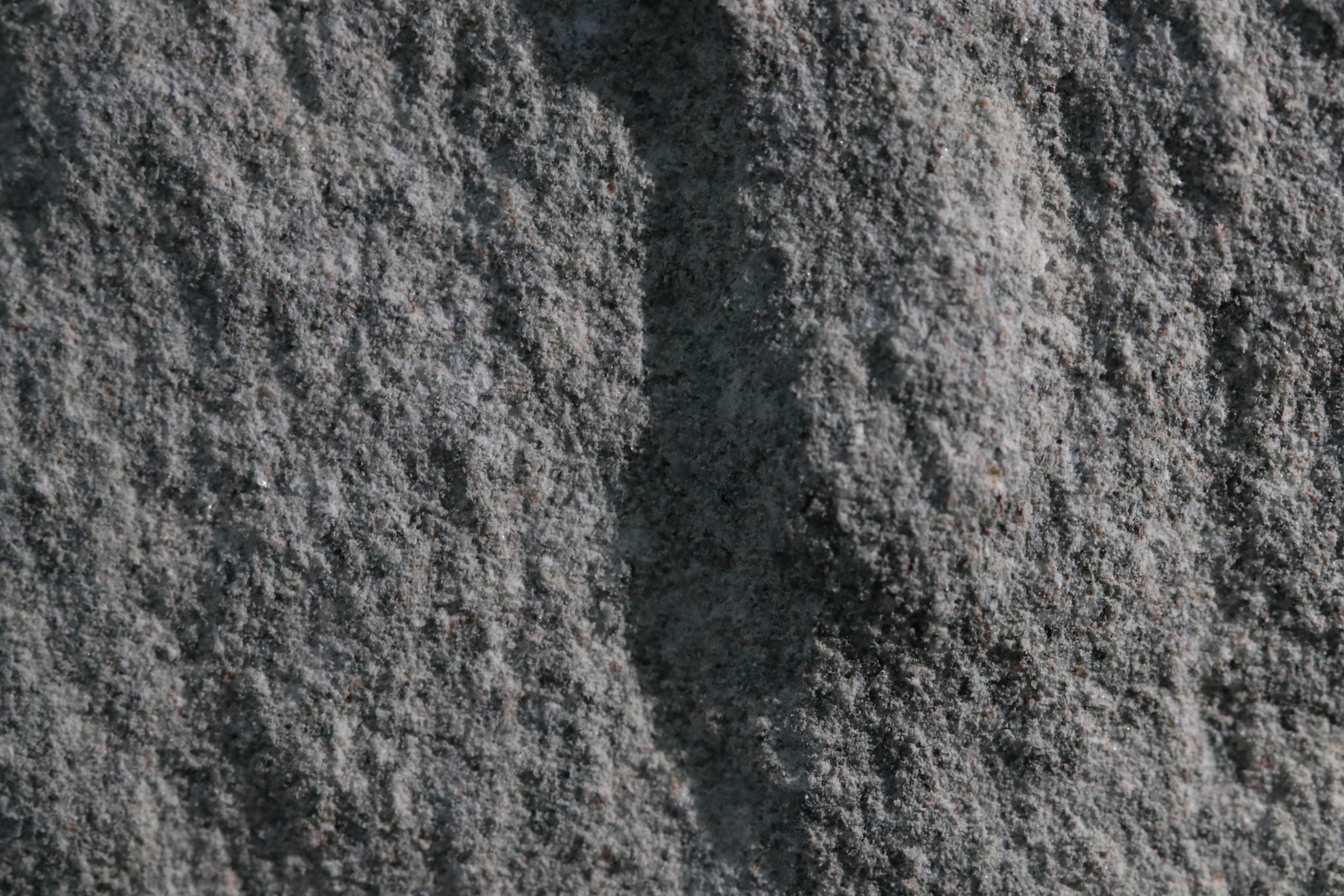

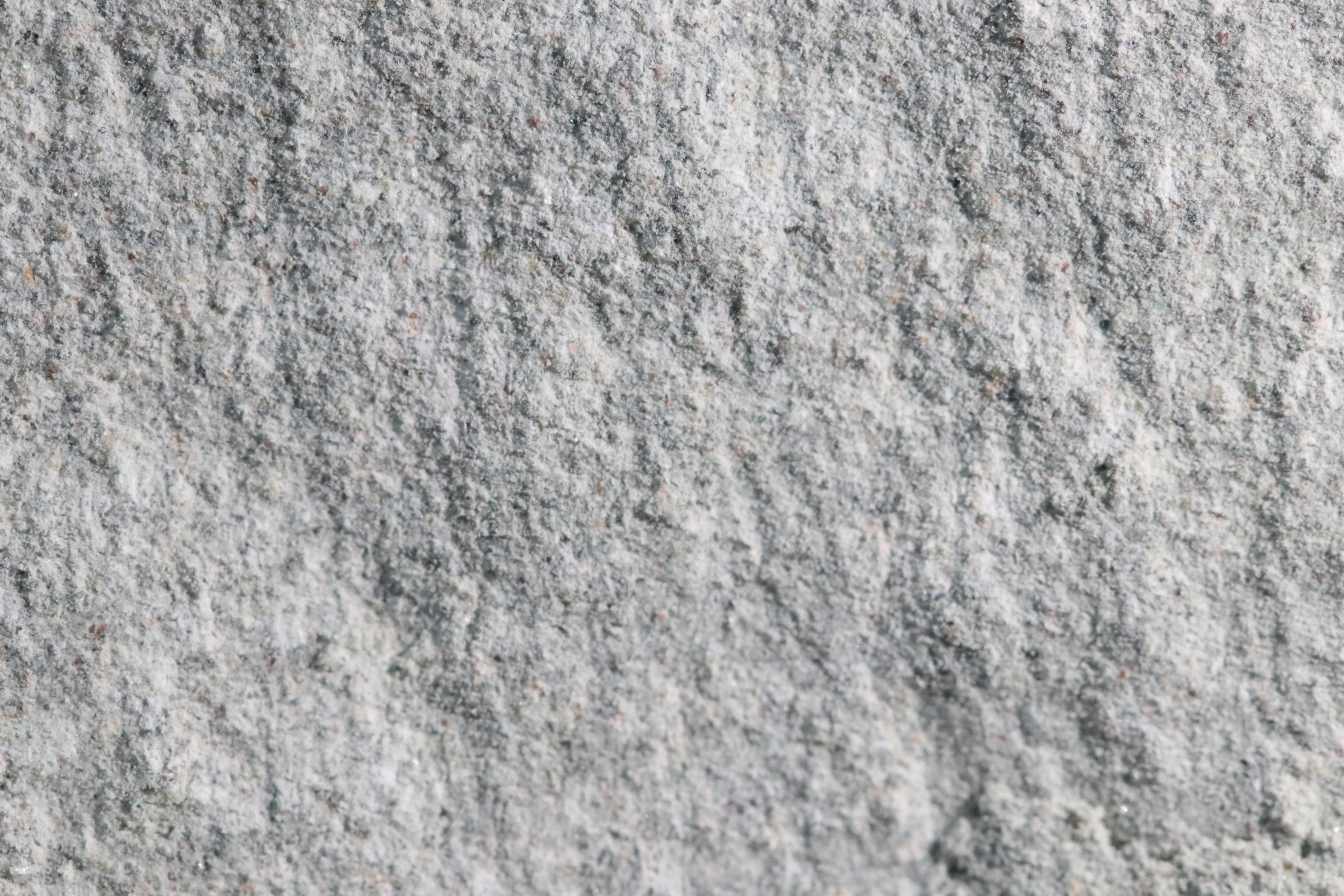

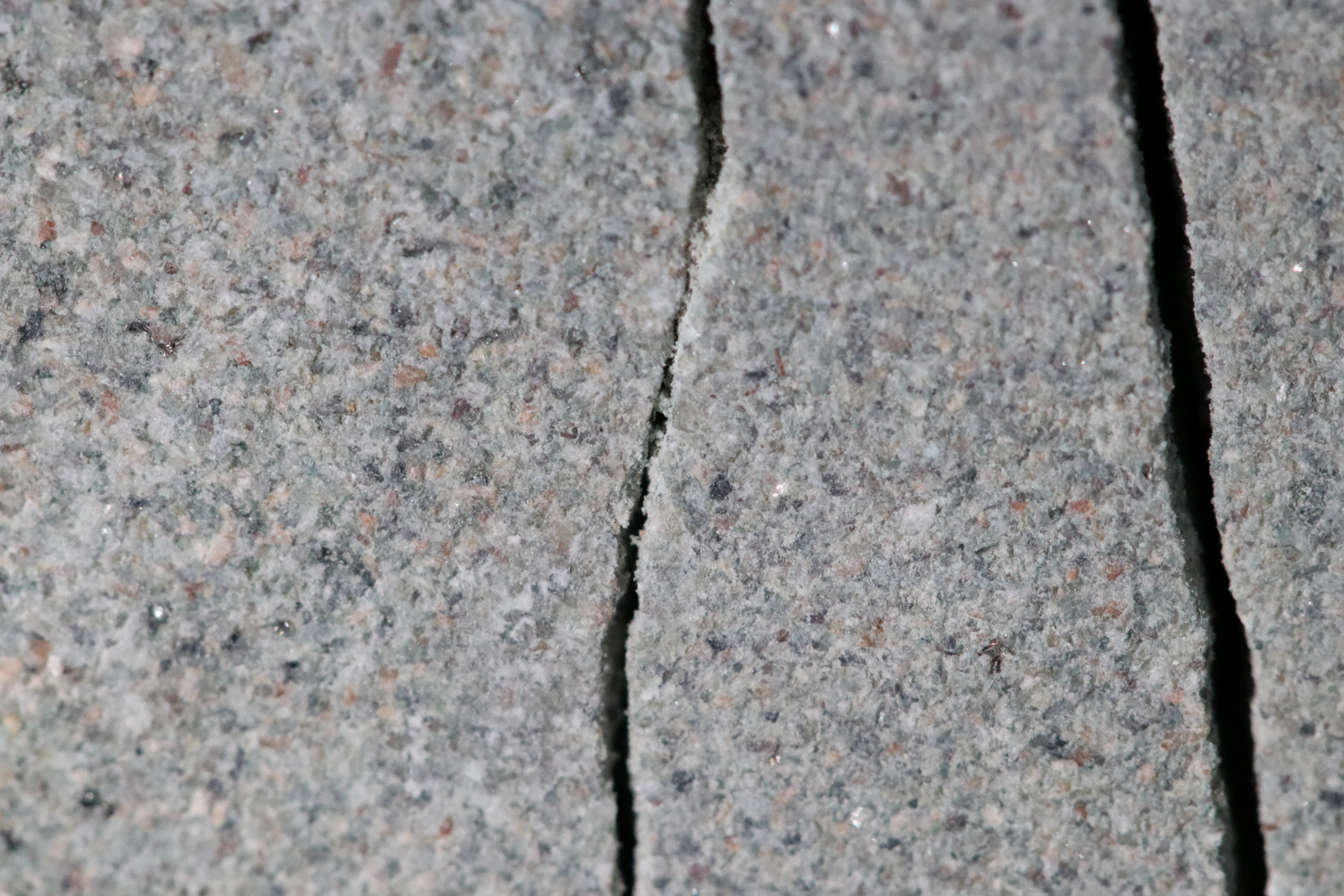

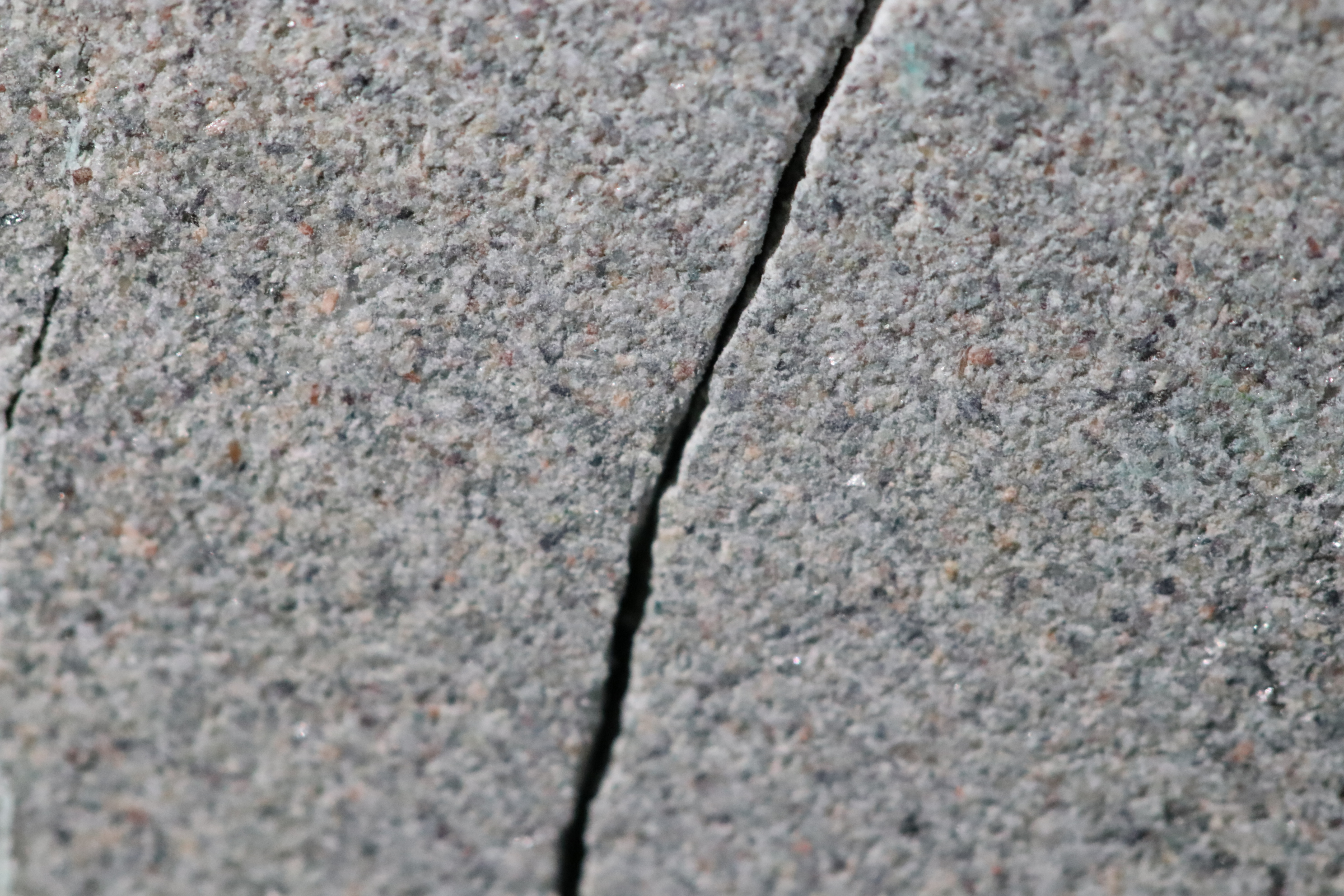

Supplement: S3 Fig — (PDF) [file pone.0323809.s003.pdf]

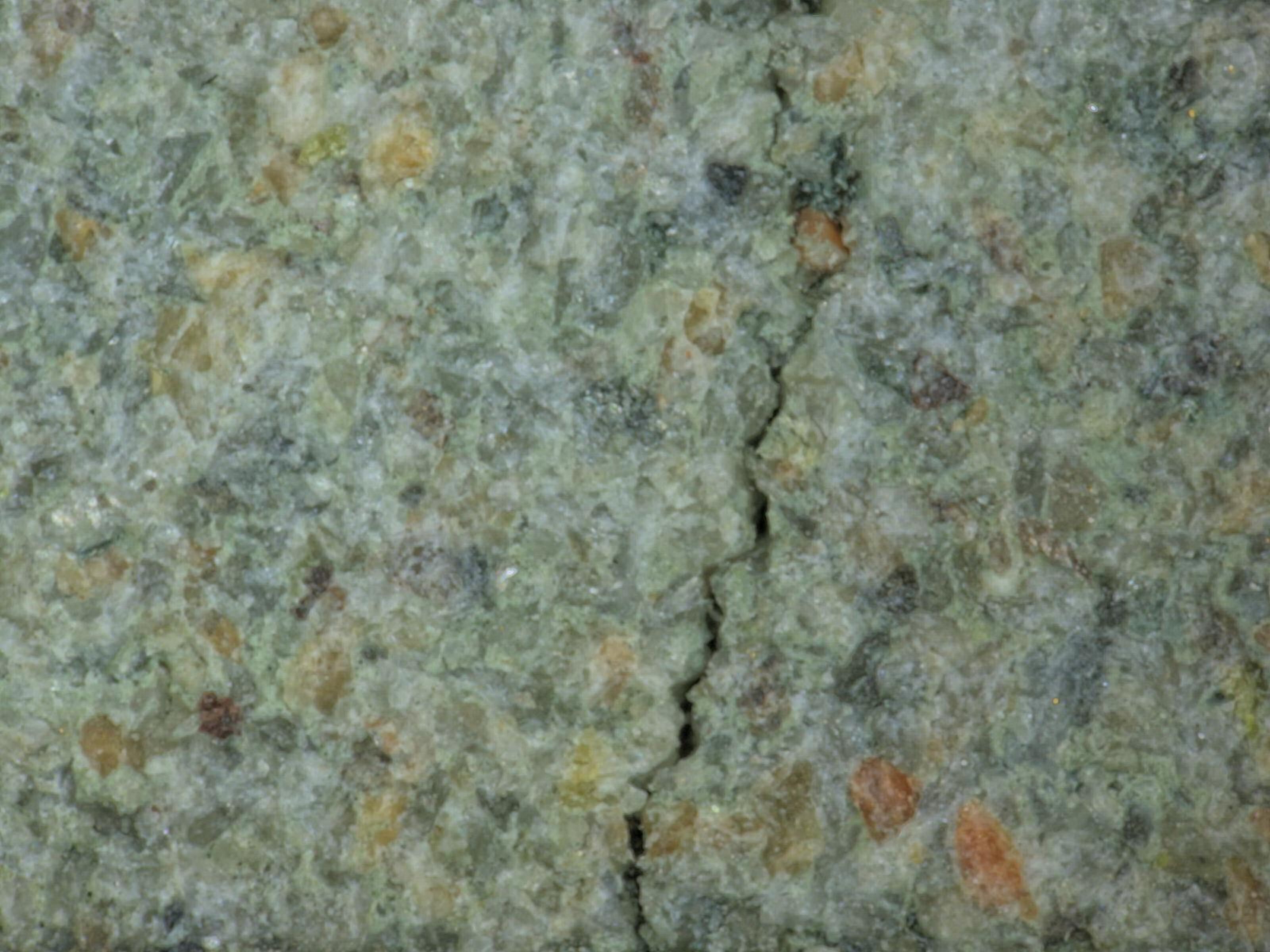

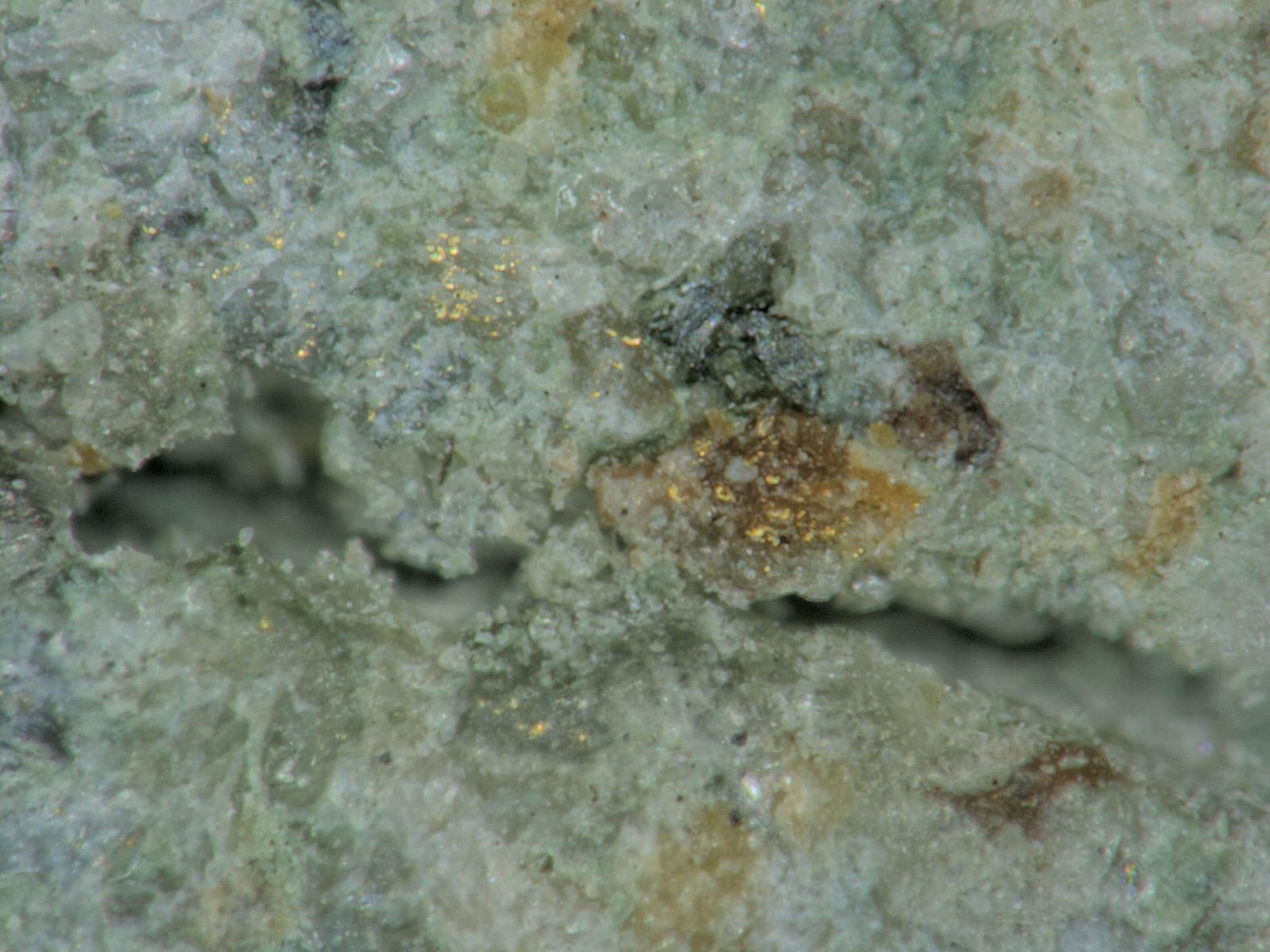

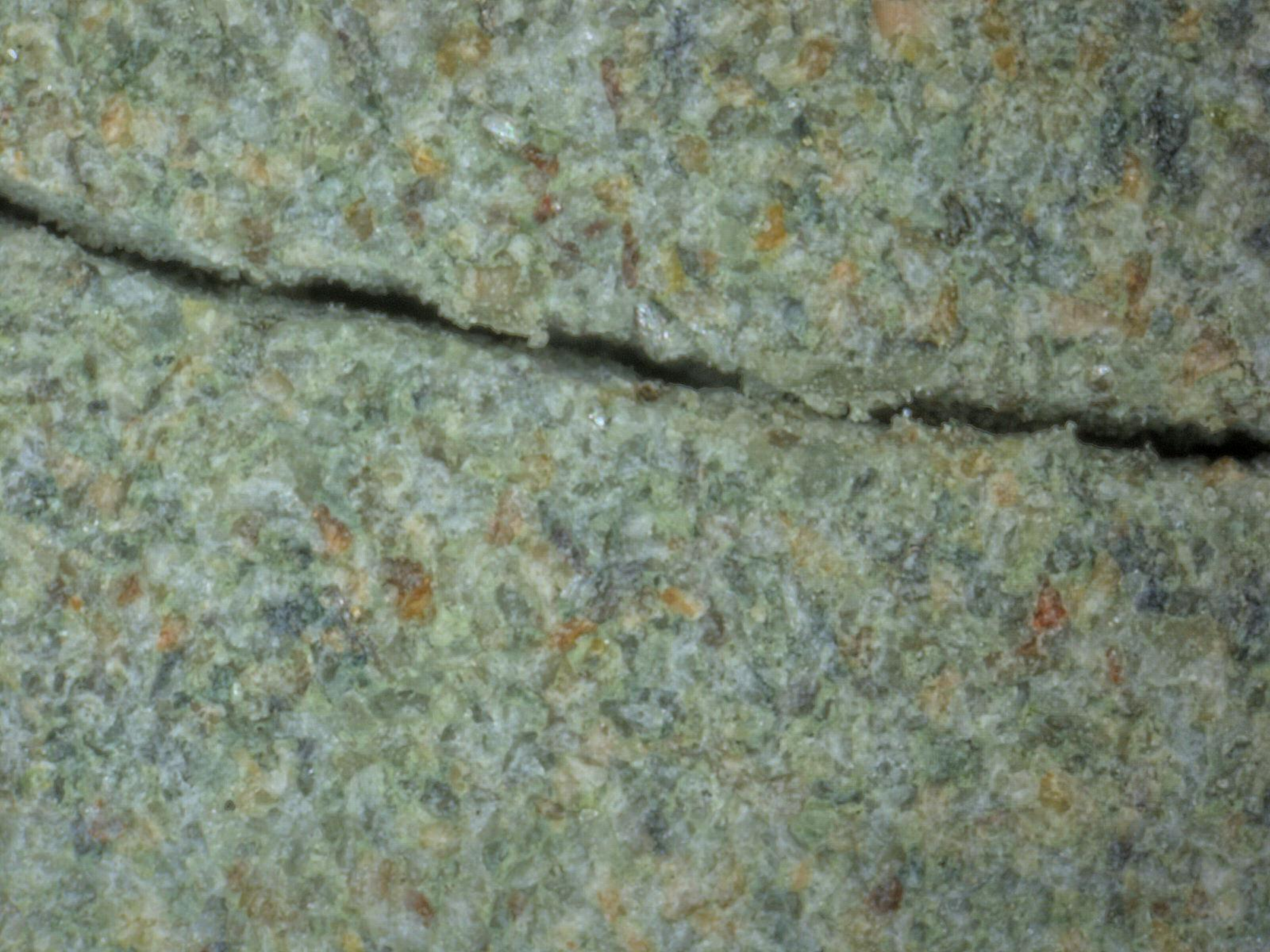

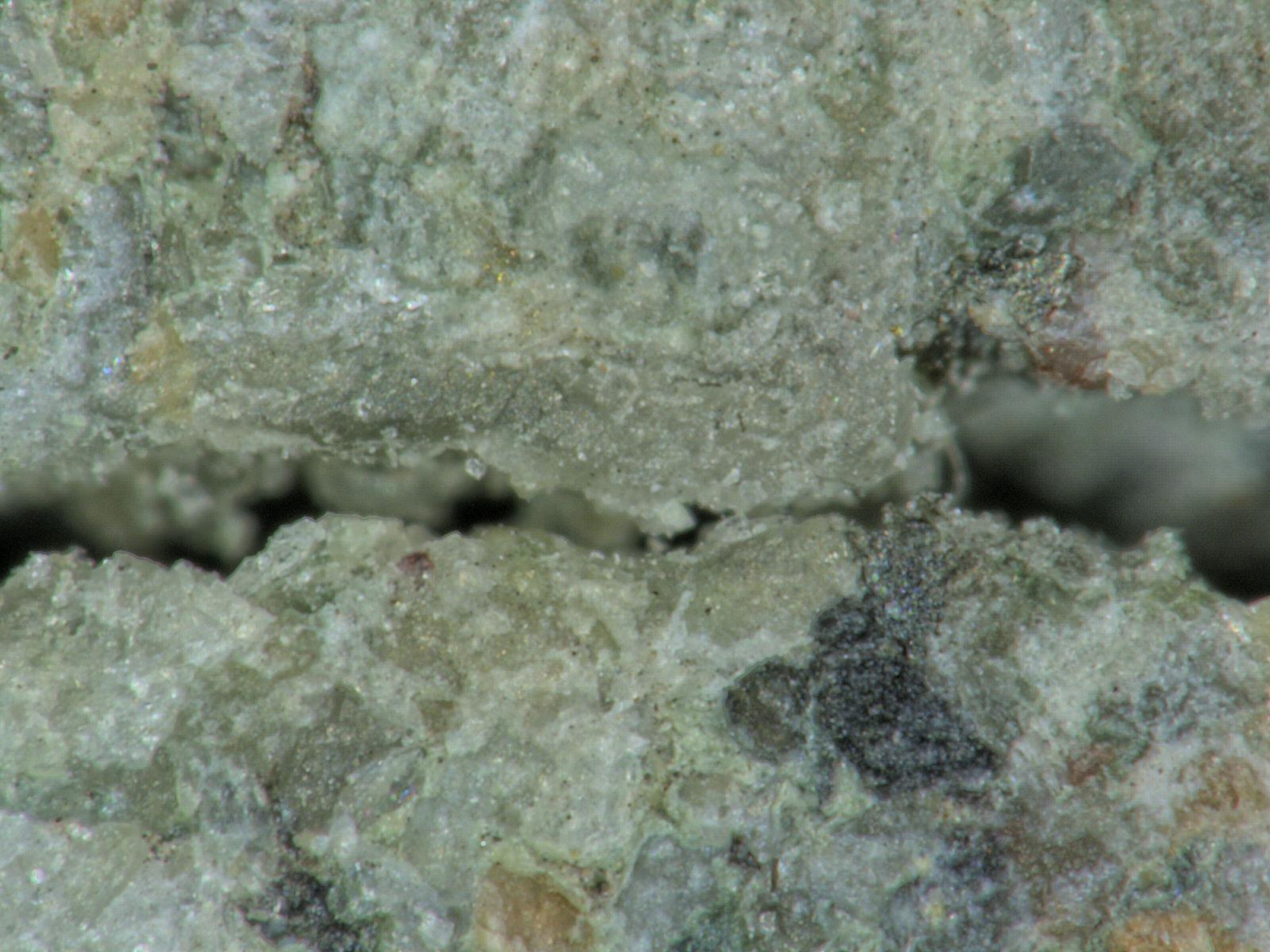

Supplement: S4 Fig — (PDF) [file pone.0323809.s004.pdf]

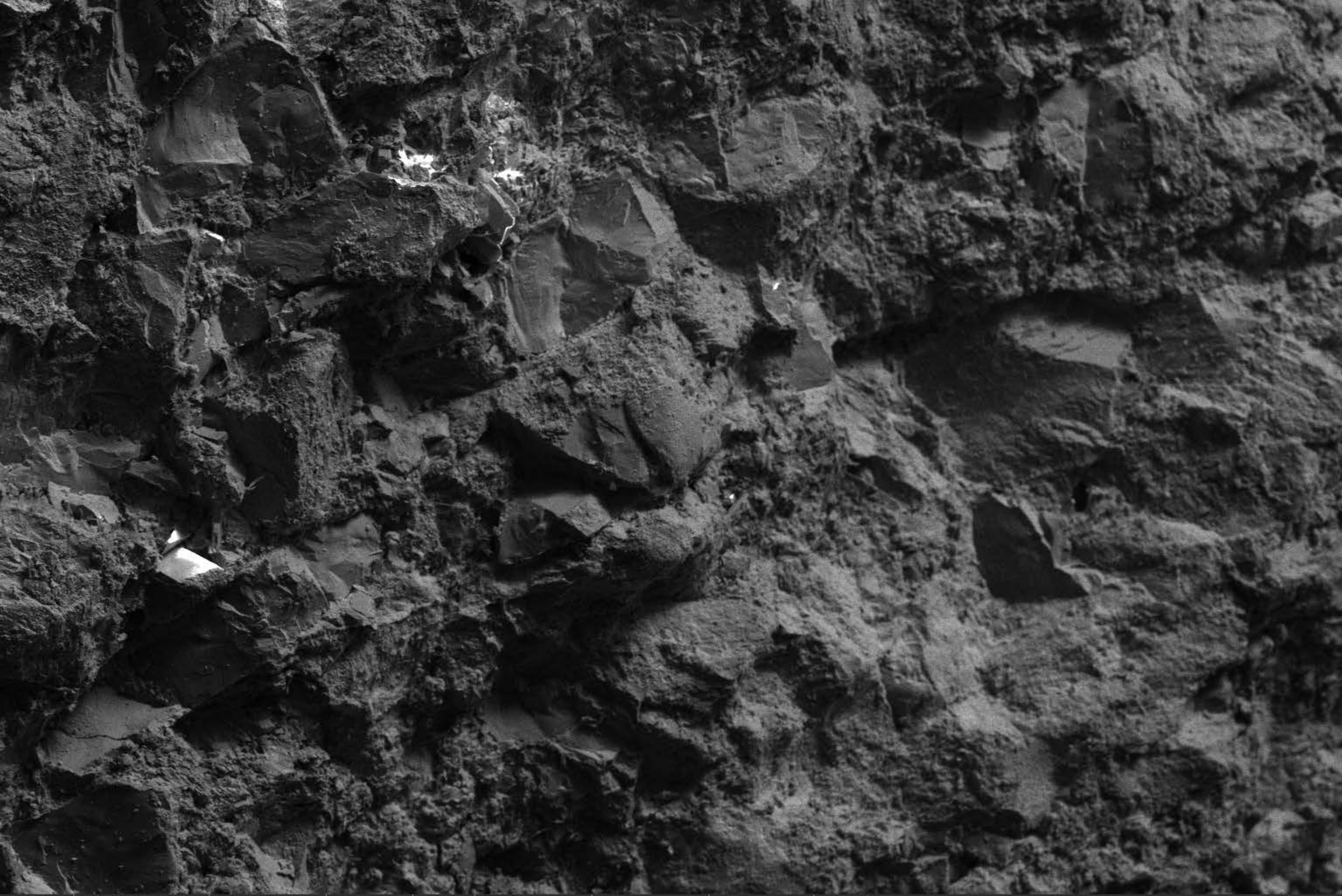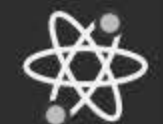

3/8/2021  
6:59:53 PM

HFW  
1.59 mm

mag 80 x

det  
ETD

HV  
10.00 kV

WD  
13.8 mm

curr  
0.74 nA

500  $\mu$ m

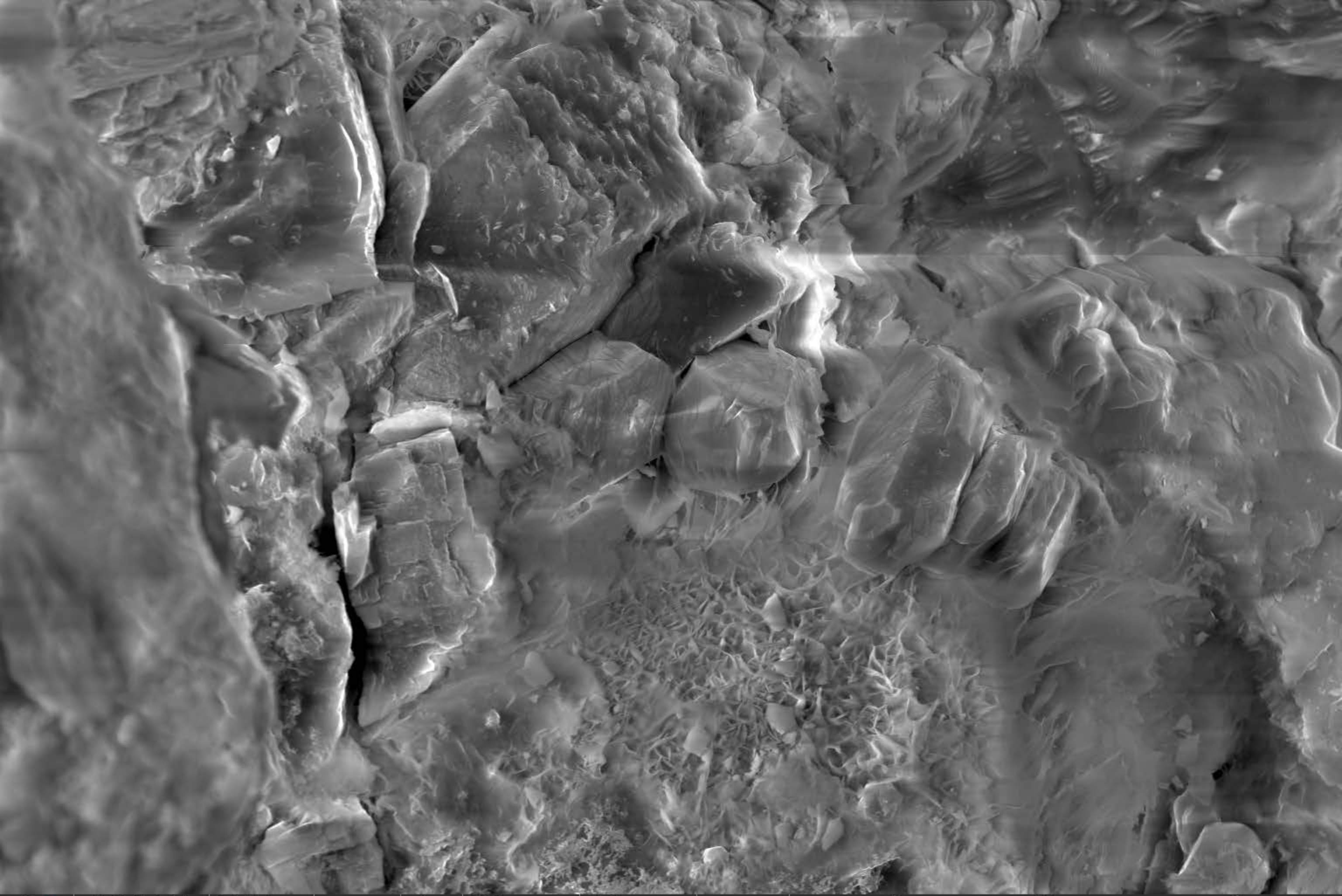

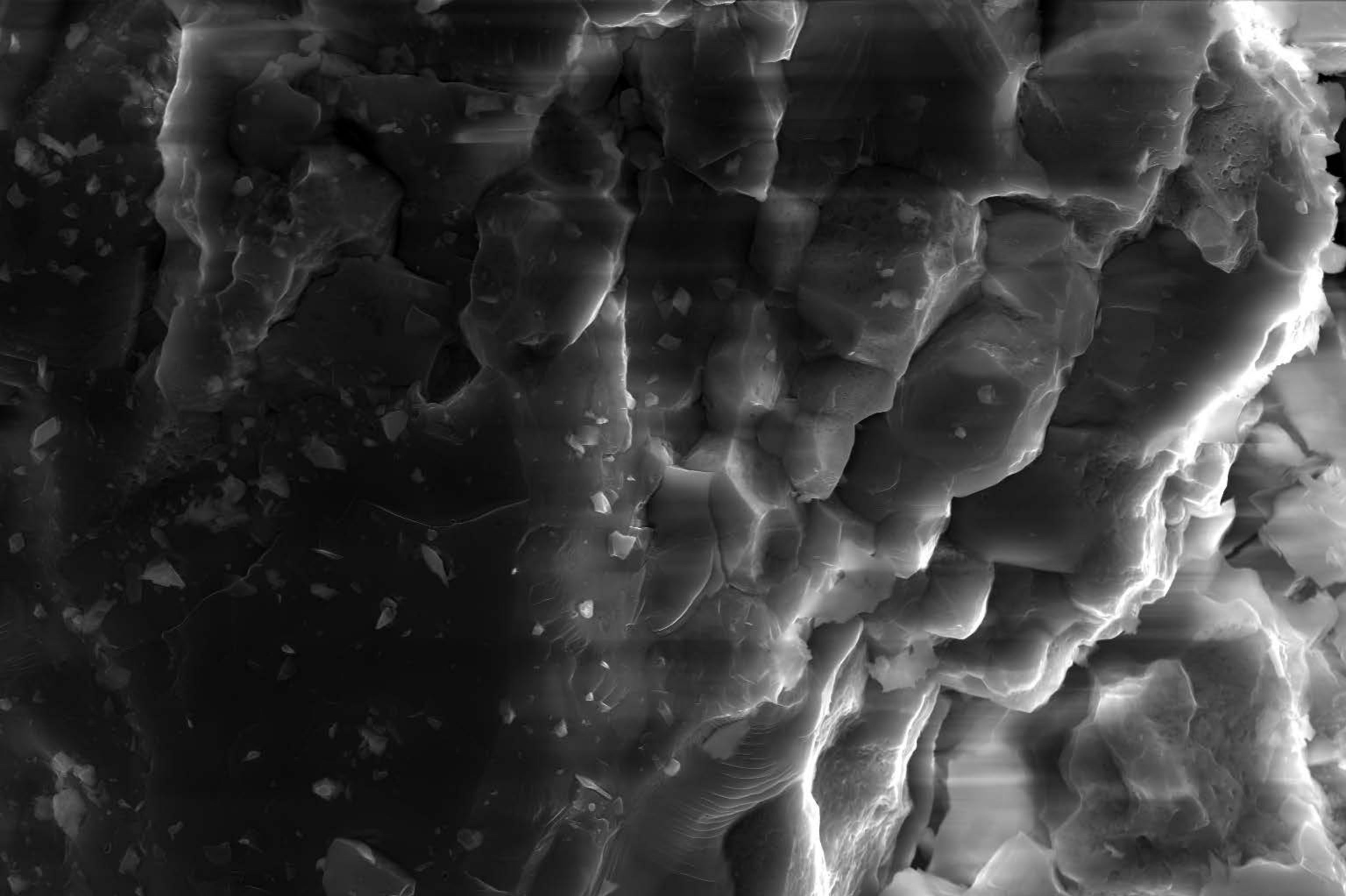

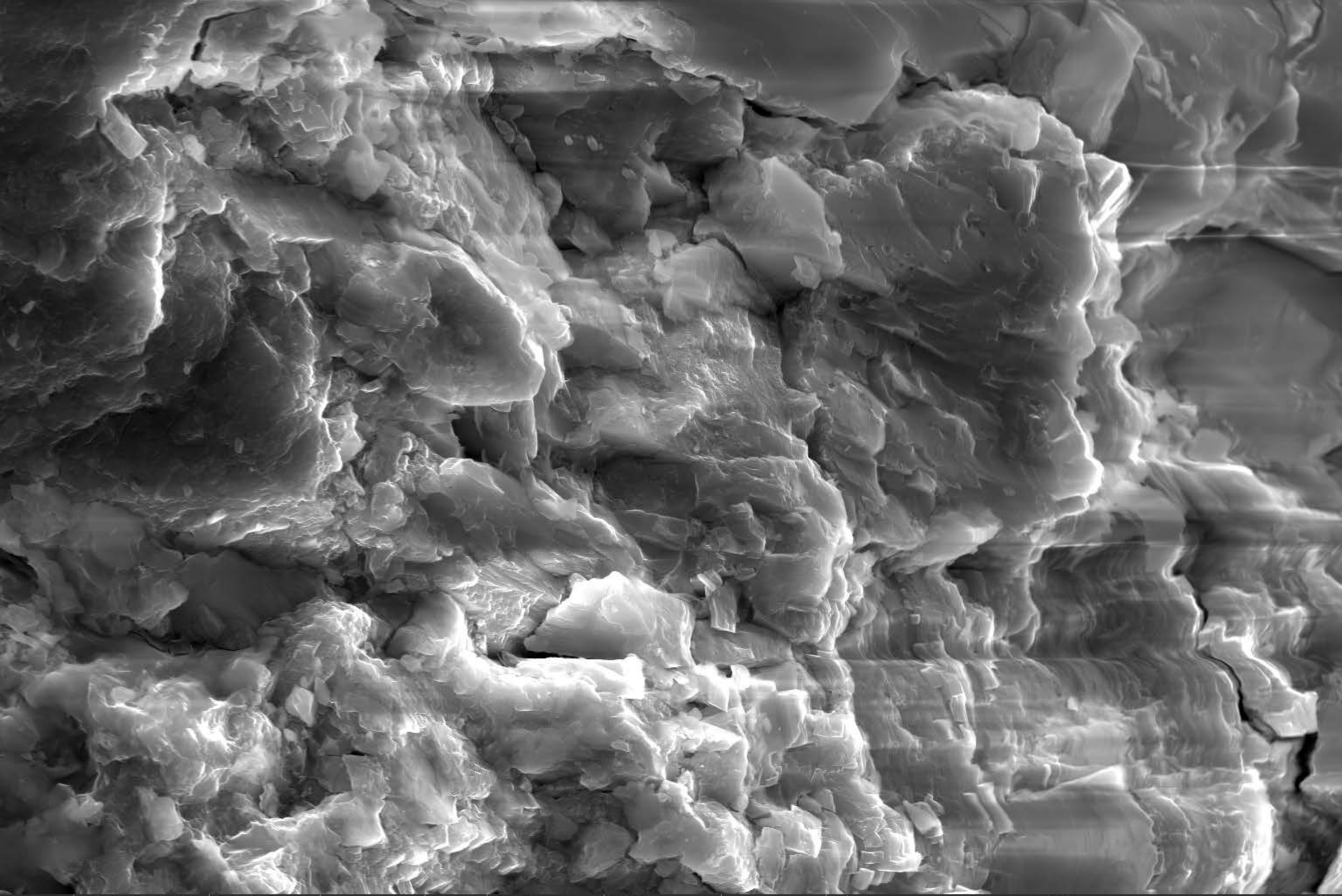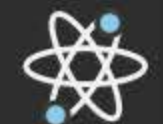

3/8/2021  
7:30:29 PM

HFW  
**84.7  $\mu\text{m}$**

mag 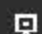  
**1 500 x**

det  
**ETD**

HV  
**30.00 kV**

WD  
**15.8 mm**

curr  
**0.31 nA**

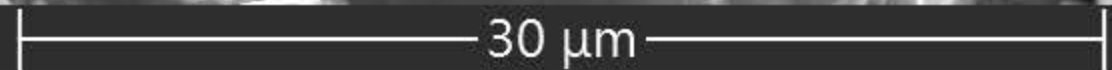 30  $\mu\text{m}$

Supplement: S5 Fig — (PDF) [file pone.0323809.s005.pdf]

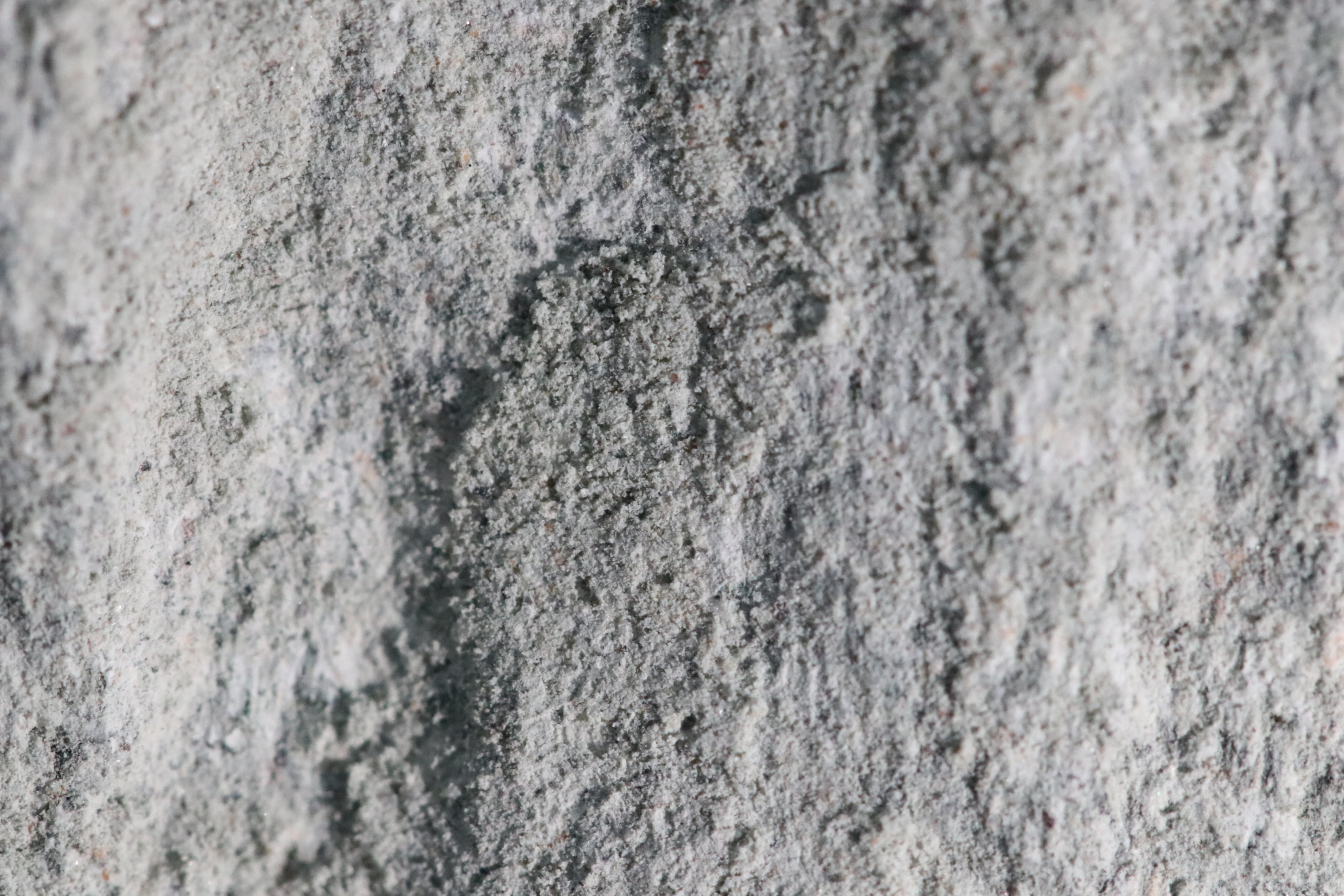

Supplement: S6 Fig — (PDF) [file pone.0323809.s006.pdf]

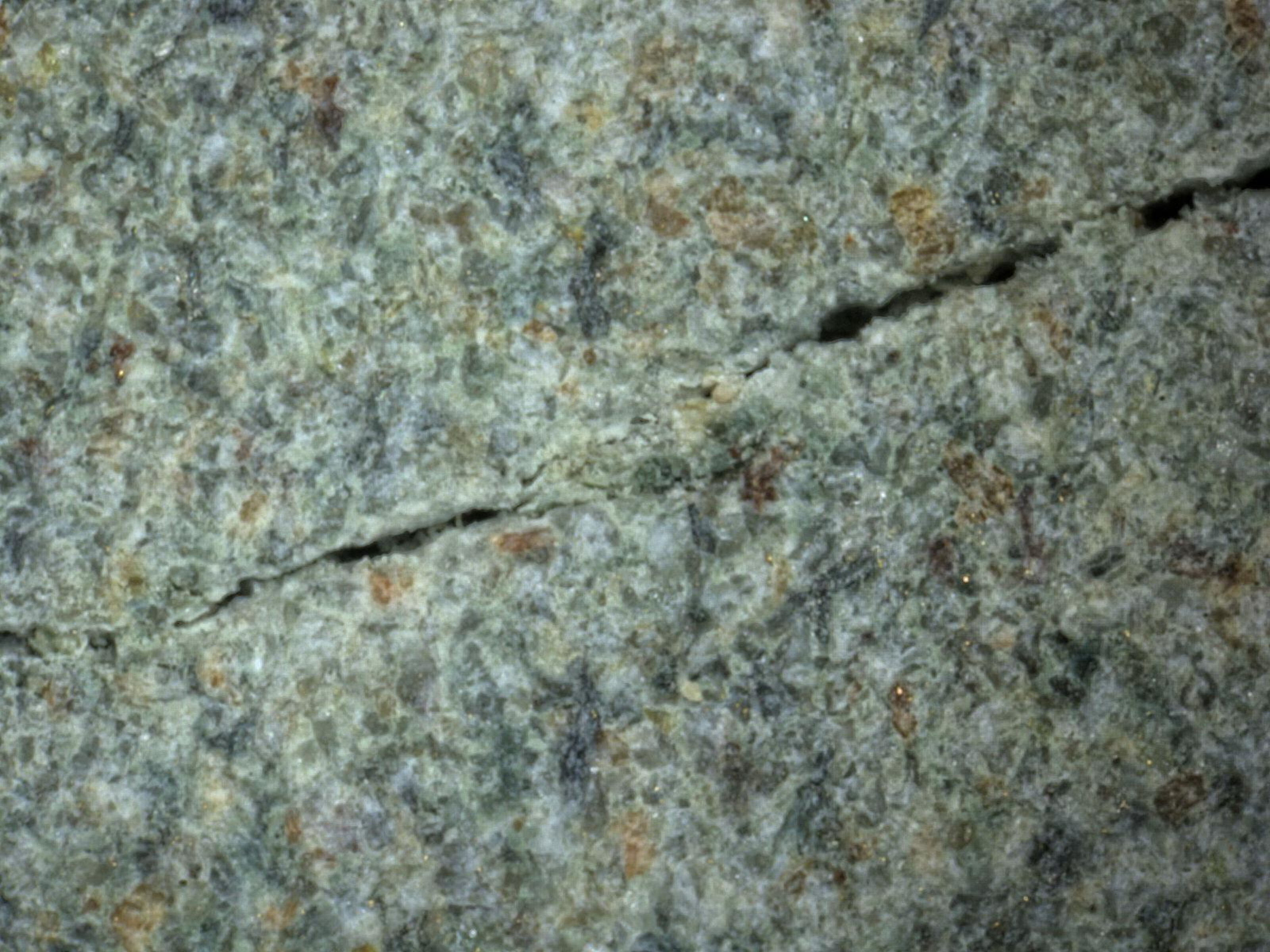

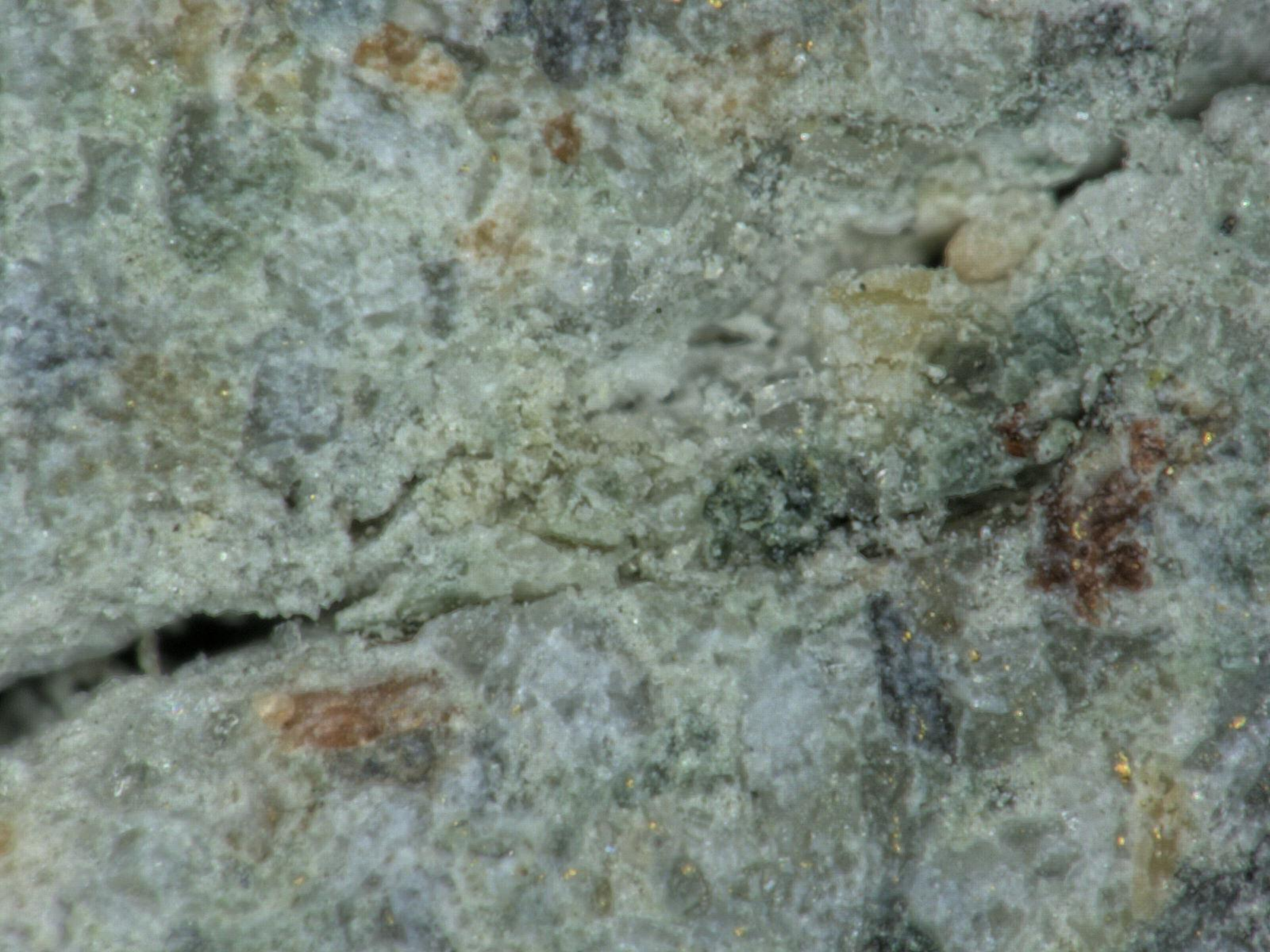

Supplement: S7 Fig — (PDF) [file pone.0323809.s007.pdf]

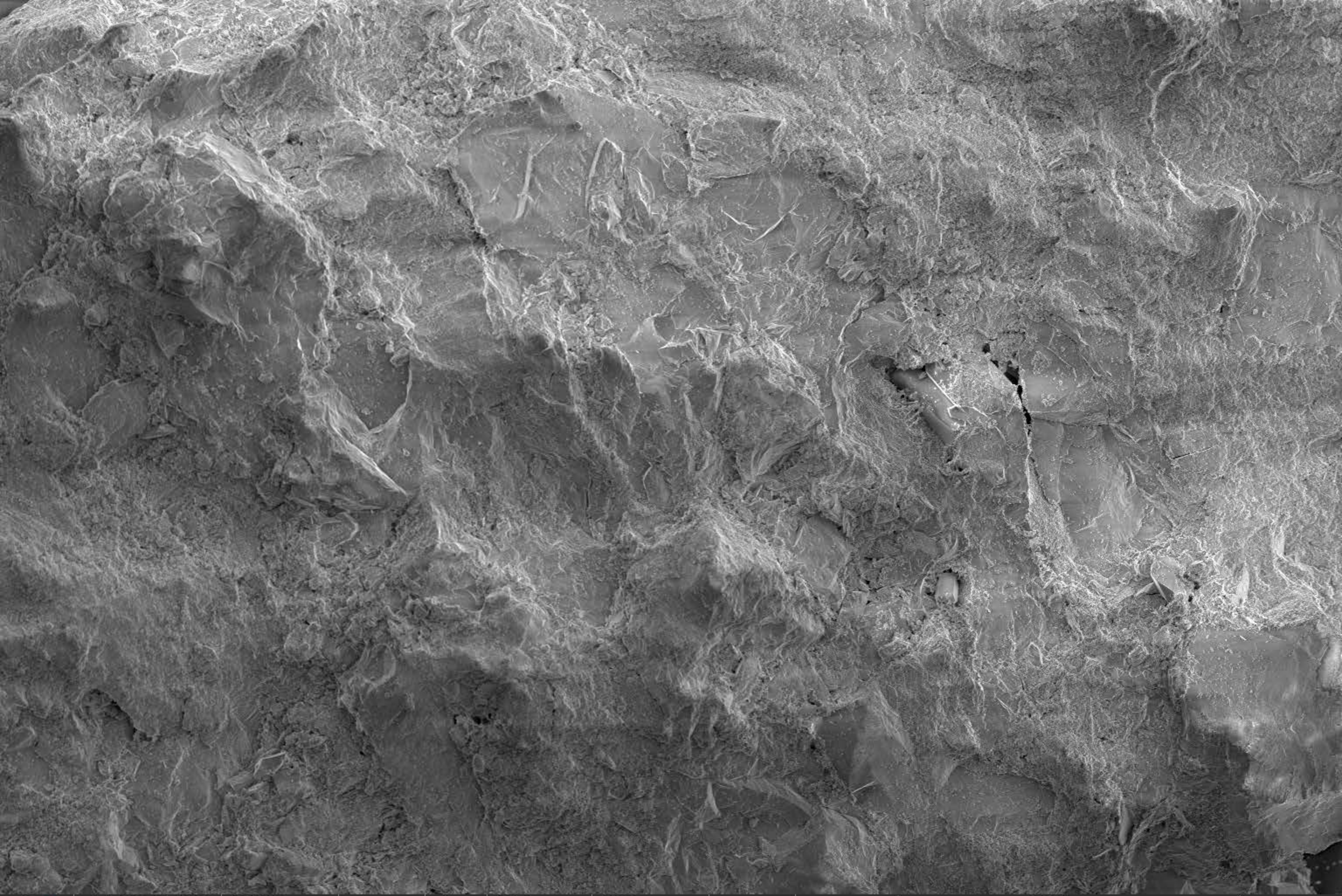

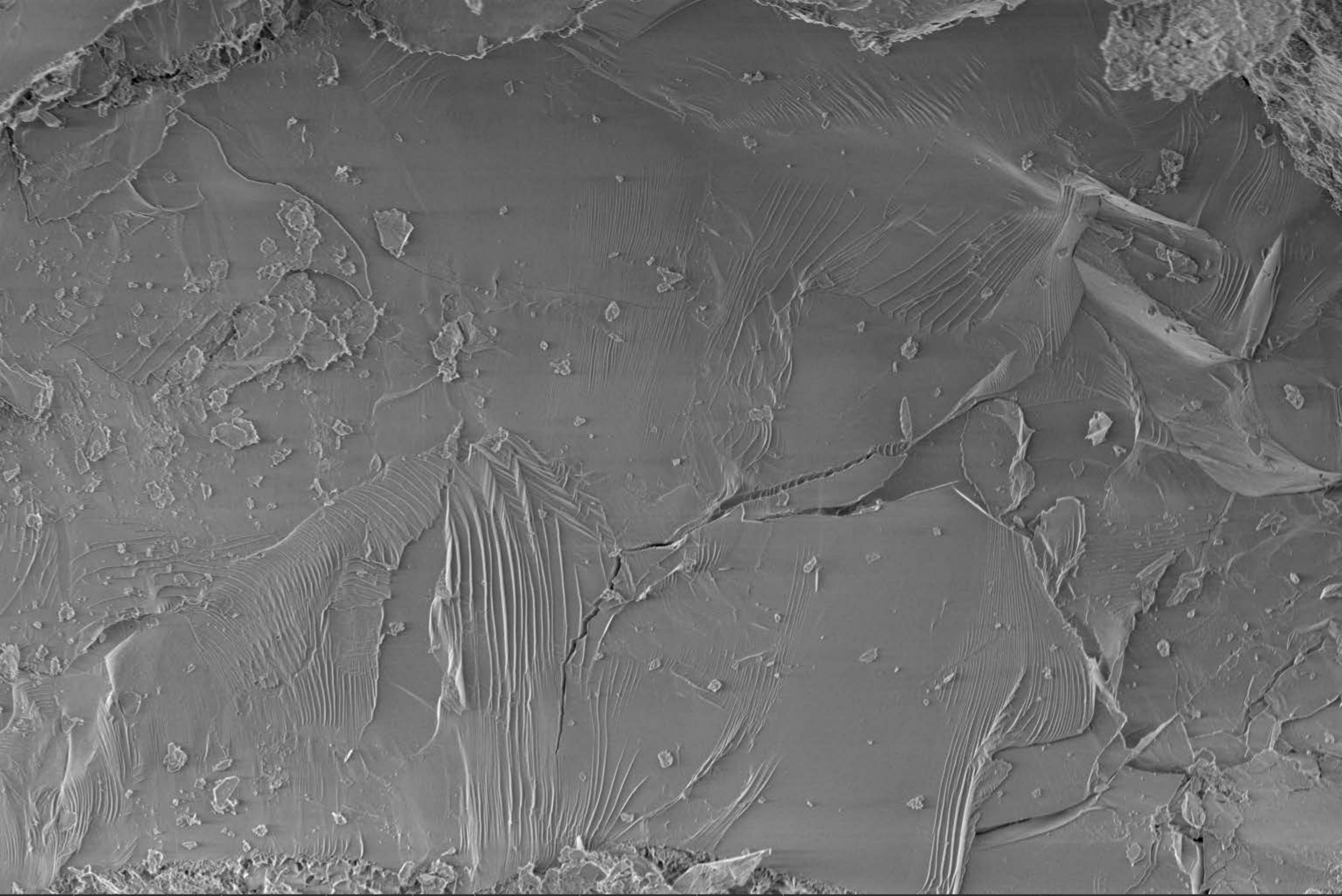

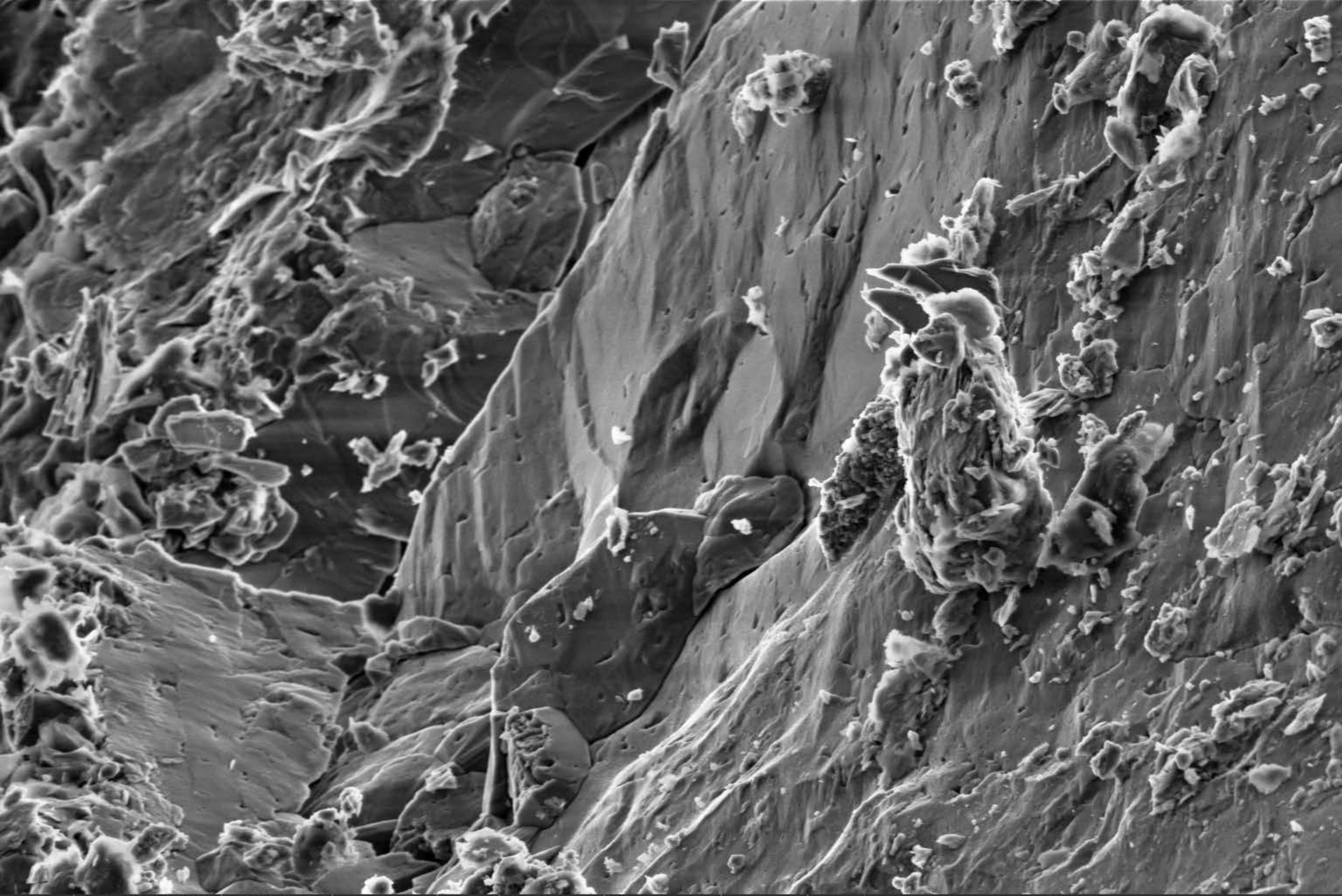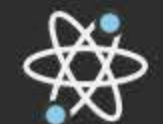

11/13/2020  
3:18:35 PM

HFW  
63.5  $\mu\text{m}$

mag 2 000 x

det  
ETD

HV  
5.00 kV

WD  
35.7 mm

curr  
57 pA

20  $\mu\text{m}$

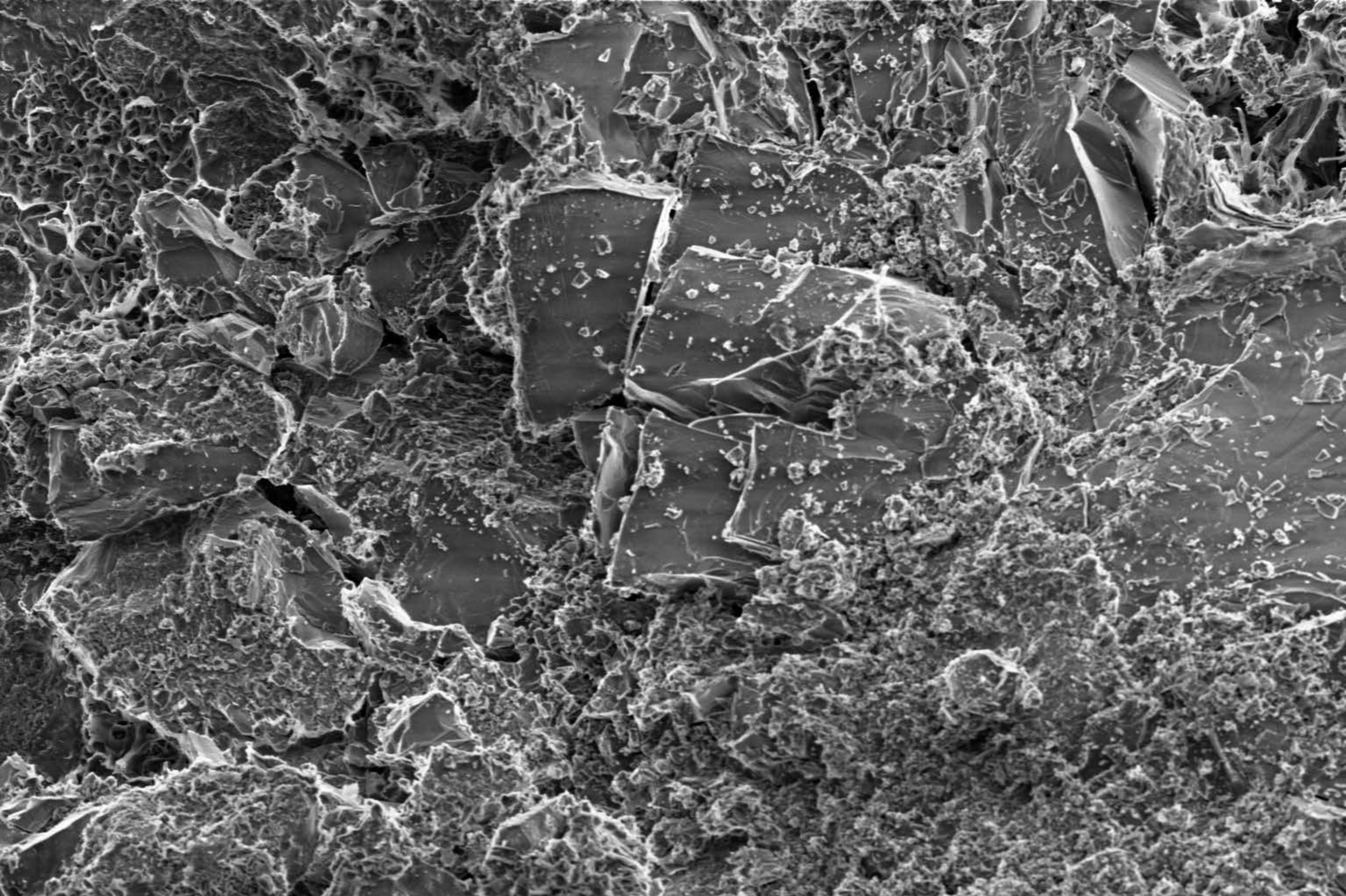

Supplement: S8 Fig — (PDF) [file pone.0323809.s008.pdf]
